# Planetary Health Diet Compared to Dutch Dietary Guidelines: Nutritional Content and Adequacy

**DOI:** 10.3390/nu16142219

**Published:** 2024-07-11

**Authors:** Julieth Pilar Uriza-Pinzón, Femke Fleur Verstraete, Oscar H. Franco, Vicente Artola Arita, Mary Nicolaou, Yvonne T. Van der Schouw

**Affiliations:** 1 Department of Global Public Health & Bioethics, Julius Center for Health Sciences and Primary Care, University Medical Center Utrecht, Utrecht University, 3508 GA Utrecht, The Netherlands; j.p.urizapinzon@umcutrecht.nl (J.P.U.-P.);; 2 Division of Human Nutrition and Health, Wageningen University & Research, 6708 WE Wageningen, The Netherlands; 3 Department of Public and Occupational Health, Amsterdam University Medical Center, University of Amsterdam, 1081 BT Amsterdam, The Netherlands

**Keywords:** healthy eating, sustainable diets, nutritional adequacy

## Abstract

In 2019, the EAT-Lancet Commission proposed a Planetary Health Diet (PHD) to address challenges toward sustainable and healthy diets. However, its suitability within the Dutch context and a comparison with the Dutch Dietary Guidelines (DDG) needs investigation. Our study aimed to compare the PHD with DDG in terms of food groups, servings, nutritional content, and adequacy in adults. We modeled two theoretical diets, the PHD (PHD-NL) and another based on the DDG (DDG-NL), using the Dutch National Food Consumption Survey (FCS-2016) and Dutch Food Composition Database to calculate the nutritional content and compared it with the Dutch Dietary Reference Values (DRVs). The PHD included higher quantities of vegetables, fish, legumes, and nuts, while the DDG suggested more significant amounts of cereals, tubers, starchy vegetables, dairy, and red meat. We observed differences in macronutrient distribution; while both diets lacked sufficient vitamin D, calcium content was lower in the PHD-NL. The PHD-NL had higher levels of fiber, vegetable protein, unsaturated fats, and non-heme iron, while vitamins B2, B6, B12, and calcium were lower than the DDG-NL diet. The PHD-NL has nutritional adequacy in the Dutch context, except for vitamin D and calcium, although it is essential to be cautious with iron because of the bioavailability of non-heme iron in plant-based diets. These findings have implications for the adoption of a sustainable diet according to nutritional requirements, population health status, and sociocultural context, as well as compliance with specific dietary behaviors of populations.

## 1. Introduction

The interest in sustainable and healthy diets is rising due to concerns about planetary sustainability and major health issues associated with current eating habits [1,2,3,4]. As a result, The Lancet published the Planetary Health Diet (PHD) in 2019 to promote human health and environmental sustainability [2]. Furthermore, since the 20th century, many governments have developed dietary guidelines to promote health, and their evolution reflects the ongoing effort to promote healthier diets and sustainability [5,6]. According to the last, some studies have compared local dietary guidelines with the PHD proposal. For instance, in Germany, there was a broad agreement between both recommendations, with the main differences in milk and dairy products [7]. Also, some researchers have developed local indexes or scores to measure the adherence of their populations to the PHD [8,9,10,11,12,13,14].

Until now, dietary guidelines have been developed with a health focus, as sustainability was not a top priority and was challenging to quantify [15]. The development of PHD followed an increased understanding of planetary boundaries and sustainability. In terms of nutritional content, the EAT-Lancet Commission reported that only vitamin B12 is low in the PHD [2]; however, some criticism around its nutritional adequacy has arisen. Authors have reported that adhering to the PHD could cause some micronutrient shortfalls in adult populations from Denmark, Italy, Australia, France, and the United States such as vitamin D, vitamin B12, calcium, zinc, iron, iodine, magnesium, potassium, fiber, and selenium [16,17,18,19,20,21].

The reported differences between the studies are due to local diets and dietary recommendations, as both are country-specific. Additionally, diverse populations may have unique dietary patterns, including preferences, restrictions, and traditional food practices, which could impact the diet’s feasibility, acceptability, and nutritional adequacy [22,23]. Translating and comparing the Planetary Health Diet in the Dutch context can help address the gaps related to its application and adaptation while ensuring it meets nutritional adequacy in the Netherlands.

Hence, in the current study, we aim to achieve the following two objectives: (1) to compare the Dutch Dietary Guidelines (DDG) and the PHD based on recommended foods with their servings and (2) to determine their nutritional content and adequacy in relation to the Dutch Dietary Reference Values (DRVs) for the adult population. We hypothesize that the Dutch Dietary Guidelines (DDG), which incorporate sustainability aspects, will resemble the Planetary Health Diet (PHD) in most aspects and also that both diets could meet the Dutch Dietary Reference Values (DRVs) for the adult population.

## 2. Materials and Methods

**Figure 1:**
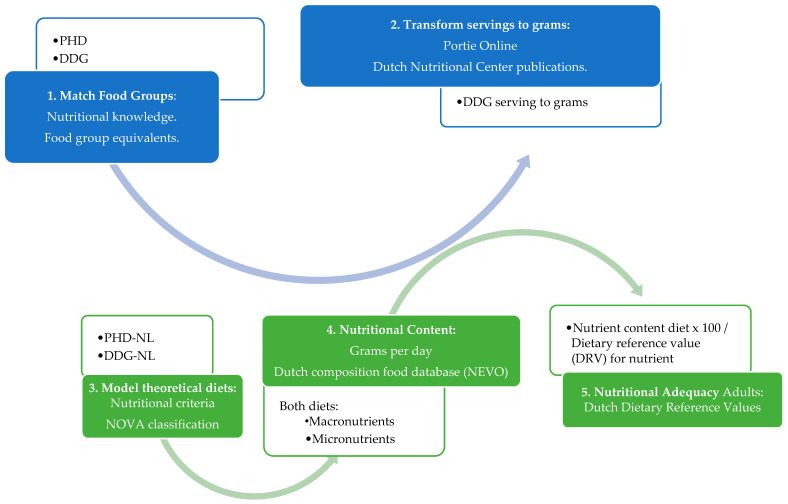
Overview of methodological steps used for the comparisons, nutritional content, and adequacy.

Our methodology is summarized in Figure 1. We follow the next steps.

### 2.1. Planetary Health Diet and Dutch Dietary Guidelines Comparison

Two recommended dietary patterns were compared: the PHD and the DDG.

- (Step 1) Match food groups: For both the PHD and the DDG, food groups were categorized according to food nature and compared to each other (Table 1);
- (Step 2) Transform serving to grams: Food groups from DDG were converted from servings to grams (g) per day through the website portie-online (https://portie-online.rivm.nl/ accessed on 16 May 2023). Additionally, the amount of bread, whole grain, nuts, cheese, fish, meat, legumes, eggs, and fats was confirmed by the publications of the Dutch Nutrition Center [24,25,26]. PHD recommendations are published in grams/day (Table 1).

### 2.2. Model Theoretical Diets

To determine the nutritional content and adequacy of these recommendations, two theoretical diets were modeled: the Planetary Health Diet adapted with Dutch foods (PHD-NL) and the healthy diet based on the Dutch Dietary Guidelines (DDG-NL).

- (Step 3) Model theoretical diets: Data from the Dutch National Food Consumption Survey 2016 (FCS) [27] were used as a source of food consumption data. Then, the NOVA (Portuguese: nova classificação, “new classification”) classification [28] and nutritional criteria were used to define the food processing level and to assign foods to food groups for both diets; (Supplementary Material S1–S4 and S6)
- (Step 4) Nutritional content: Nutritional content was analyzed using the grams per day for every food, with the Dutch food composition database (NEVO) [29], summed up, and then averaged for every food group; (Supplementary Material S7)
- (Step 5) Nutritional adequacy: Finally, the total nutritional values of each diet were compared with the Dutch Dietary Reference Values (DRVs) for adults from 18 to 50 years [30,31]. For calcium DRVs, an average was calculated. (Supplementary Material S5)

Comparisons were made with the estimated average requirement (EAR) or, when this was not available, the Adequate Intake (AI). All attributions of products to food groups and calculations were performed using IBM SPSS Statistics 26 for Windows version 26.0.0.1.

## 3. Results

### 3.1. Food Groups Comparisons

The PHD and DDG differ in some characteristics, like food categorization, measurement units, amounts, and flexibility parameters. The PHD contains eight food groups and fourteen subgroups, and the DDG has five food groups and eight subgroups. Concerning the measurement units, the PHD reports specific food amounts in grams and the DDG in a range of servings. Also, the amount of some animal-source foods, like dairy foods and red meat, was higher in the DDG diet. Finally, in terms of flexibility, the DDG allows the consumption of products that are not advised by the Health Council (i.e., 15% of total daily energy intake can come from products that fall outside the five food groups that are advised), whereas the PHD limits energy intake within the recommended groups.

The results indicate that there are some quantitative differences between the PHD and the DDG. The PHD recommends lower intakes, in grams per day, than the DDG for cereals, whole grains, tubers, starchy vegetables, dairy foods, red meat, and eggs. Moreover, the PHD has higher intake recommendations for vegetables, chicken, fish, legumes, and nuts. In the fats group, when differentiated by sex, PHD recommendation is higher for women and lower for men.

The PHD does not distinguish cheese from other dairy products, but the DDG does. The PHD includes added sugars as food groups, whereas the DDG does not, and the PHD does not include recommendations about beverage intake, whereas the DDG does. Thus, for added sugar and drinks, there was no equivalent group to compare both dietary patterns.

### 3.2. Nutritional Content and Adequacy

The average micro- and macronutrient content of both diets, as recommended, is presented in Table 2 and Table 3, together with the corresponding percentage of the Dutch Dietary Reference Values. The energy content of the two diets is similar (PHD-NL 1952 kcal and DDG-NL 1968 kcal). However, the nutrients from which this energy is derived differ between the two diets. In PHD-NL, most of the energy comes from fat, while in the DDG-NL diet, most of the energy comes from carbohydrates. 

In the DDG-NL diet, 44% of energy is obtained from carbohydrates, while in the PHD-NL diet, it is 37%, and both percentages are around the recommended level of 40% of energy. Part of the carbohydrates are mono- and disaccharides, of which the levels are also higher in the DDG-NL diet than in the PHD-NL diet. On the contrary, the levels of polysaccharides and fiber are higher in the PHD-NL diet than in DDG-NL. For the PHD-NL, the amount of fiber (39 g) is approximately in line with the DRVs for both males and females, while the DDG-NL contains 30 g of fiber and is therefore in line with the recommendation for females but not with the recommendation for males.

**Figure 2:**
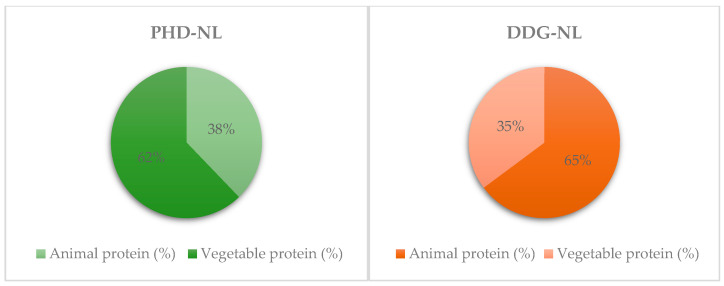
Protein distribution between animal and vegetable proteins in the theoretical diets modeled. PHD-NL: theoretical diet based on Planetary Health Diet from EAT-Lancet Commission. DDG-NL: theoretical diet based on Dutch Dietary Guidelines.

In both diets, the percentage of energy from protein is in line with the DRV and below the recommended upper limit of 25%. However, the source of protein differs between the two diets. In the PHD-NL, most of the protein (62%) is from vegetable sources, while in the DDG-NL, 65% of protein intake is from animal-source foods (Figure 2).

Energy from fat is higher in the PHD-NL than in the DDG-NL, mainly because of polyunsaturated fat. Also, the percentage of energy from polyunsaturated fat (13%) is slightly above the recommended upper limit (12%) in the PHD-NL, while the DDG-NL diet contains 11%. For saturated fat, both diets are in line with the upper limit (10%) as they contain 9.9% (PHD-NL) and 8.9% (DDG-NL). Furthermore, the percentage of energy from trans-configured unsaturated fat is below the upper limit (1%) for both diets. For specific fatty acids, both diets are in line with most of the DRVs; however, the DDG-NL diet contains only 146 mg of marine fatty acids and is below the recommendation.

For most vitamins and minerals, the content of both diets is quite similar, but some differences were found in vitamin D, calcium, selenium, and potassium. The amounts of vitamins that are present in the PHD-NL diet and the DDG-NL diet are almost completely in line with the DRVs, but the amount of vitamin D is below the DRV. Of the 10 minerals that are assessed in this analysis, the level of calcium is below the DRV in the PHD-NL diet, while the DDG-NL contains 1652 mg and meets nutritional requirements for adults. And in both diets, sodium is below the DRV.

Additionally, based on the NEVO, water content from food and beverage sources was calculated. The DDG-NL would result in almost three liters of water (2999 g), while PHD-NL resulted in one liter (1077 g).

## 4. Discussion

### 4.1. Key Findings

We aimed to assess the similarities and differences between the PHD and DDG in terms of food groups, servings, nutritional content, and adequacy in Dutch adults. We found some differences in (1) foods and food group classifications, (2) amounts per food group, (3) nutritional content, and (4) nutritional adequacy. First, regarding food classifications, there was no equivalent group to compare between added sugar included in the PHD and drinks included in the DDG. Second, in the amounts per food group, we found that PHD recommends higher amounts of vegetables, fish, legumes, and nuts, while the DDG includes higher values for cereals, tubers, starchy vegetables, dairy, and red meat. This result was similar in Germany in relation to milk and dairy product recommendations [7]. Third, when operationalized based on reported intake data from the Dutch population, the PHD-NL results in higher contents of fiber, vegetable protein, and unsaturated fats, while the DDG-NL results in higher vitamins B2, B6, B12, and calcium. And fourth, we found that the modeled diets met most of the DRVs with an exception for vitamin D in both and for calcium in the PHD-NL.

For carbohydrates, the PHD-NL is higher in fiber and lower in sugars, which could be explained by the higher amount of plant-based foods in the PHD. Also, PHD does not have an equivalent for the added sugars group in the DDG. Diets with more fiber and less sugar are healthier and contribute to the reduction in morbimortality [32,33,34,35,36,37], which is a positive aspect of the PHD and would be worth reviewing for future dietary guidelines. In the case of fat, the PHD-NL has 42% of energy intake from fats, exceeding the maximum of 40%. Despite this, PHD-NL included a higher content of polyunsaturated fatty acids above the DRV. The DDG-NL resulted in half of the polyunsaturated fats and did not reach the recommendations. This can be explained by the fact that the PHD recommends higher intakes of fish and nuts than the DDG. There is evidence that polyunsaturated fats from fish and nuts have positive effects on health [32,38,39,40]; thus, higher consumption could contribute to more sustainable and healthier diets [2].

In terms of protein, both diets were above the DRV, but they differed in their protein source. As expected, DDG-NL has less vegetable protein than PHD-NL, which aligns with their food recommendations. The DDG recommends more red meat, while the PHD recommends more vegetables, legumes, and nuts, which results in higher vegetable proteins and non-heme iron in the PHD-NL. Non-heme iron in the PHD-NL is an aspect to consider during its application because protein and iron availability are generally better in animal sources [41,42,43,44]. Indeed, Beal et al. found that PHD falls short of the iron requirements (55%) for women aged 15–49 years [20], which is essential in women of reproductive age [45]. Thus, populations like fertile women could require additional adjustments in the diet for iron adequation [46], also considering the growing food insecurity and malnutrition rates among women compared to men in some populations [47].

Our results in micronutrient adequacy are consistent with some previous research examining the EAT-Lancet diet. In an Italian study, it was found that the PHD-IT diet contained 675.6 mg of calcium and 0.0019 mg of vitamin D [17,48]. In a Danish study, 684 mg of calcium and 0.0025 mg of vitamin D were found in the PHD with Danish foods, being below the recommendations [16]. Also, in Australia, PHD falls short of the calcium requirements (71%) [18]. Vitamin D intake and status is a widespread public health issue across all age groups, and our research confirms it, so addressing this problem will need further research and public policies [49,50,51]. Likewise, our results demonstrated that even with limited intake of animal-source foods, the requirements for vitamins A, B2, B6, and B12 were met with the PHD. Also, other studies in adults and women of reproductive age reported vitamin B12, calcium, iron, and zinc below recommendations [20].

### 4.2. Strenght and Limitations

A strength of this study is that it provides a complete overview of the nutritional adequacy of the PHD-NL diet and DDG-NL diet and the dietary intake of the Dutch adult population. Another strength of this study is the use of the National Food Consumption Survey (FCS) and the Dutch food composition database (NEVO). In this FCS, 91.9% of the participants had a Dutch background, and 8% were from other ethnic groups [27]. As migration is growing and it could be a determinant of health [52,53,54,55,56], in further research, nutritional adequacy of PHD-NL could be assessed in first- or second-generation Dutch people with different ethnical backgrounds and dietary habits.

We had some limitations related to the inclusion, classification, or exclusion of food groups. The guidelines of the PHD from the EAT-Lancet Commission contain little detail on food nutritional composition [2]. Because of this, nutrient criteria were developed to define which products met categorization into a food group. As not all products mentioned in the FCS were in line with the nutrient criteria for being included in the PHD-NL, these were excluded. Additionally, the PHD recommendations divide fats into four groups; however, it was not possible to attribute all added fats that were consumed by the Dutch population because most of the foods have a combination of multiple fats, e.g., Dairy fats. Also, another PHD recommendation that could not be fully followed pertained to exchanging certain food items. However, only the beef, lamb, and pork subgroups were merged, as they shared the same caloric density. All of the above can result in different group classifications than the EAT-Lancet Commission intended.

### 4.3. Implications

The current study highlights deficiencies in vitamin D in both the PHD-NL and the DDG-NL, as well as in calcium in the PHD-NL. This is consistent with some authors that emphasize the need to monitor calcium intake status in vegetarian and vegan populations [57,58,59]. Therefore, a possible solution could be the development of an optimized version of the PHD with higher levels of these nutrients while considering planetary boundaries. Additionally, biofortification and fortification of plant-based foods could be a solution to balance sustainable diets with calcium and vitamin D consumption [60,61]. 

While the PHD lacks specific recommendations for beverages, the DDG addresses this gap. It could be necessary to consider water consumption within the framework of a Planetary Health Diet. Adults require an average of 2–3.7 L of water daily, as water is an essential nutrient for body hydration [62,63]. Sustainability considerations should extend to assessing future projections of water availability for hydration and its environmental implications. Moreover, there remains a knowledge gap regarding water requirements, consumption patterns, and their impacts on health and the environment [64]. Consequently, recommendations and assessments for healthy and sustainable diets should incorporate considerations for water.

### 4.4. New Direction for Future Research

Our findings provide an answer to the need for dietary approaches that account for diverse contexts and populations, as advocated by the World Health Organization [65]. This requires revisiting food-based dietary guidelines (FBDG) in each country. Recent analysis has revealed that a significant portion of FBDG worldwide is incompatible with both the agenda on non-communicable diseases and the Paris Climate Agreement [15]. Thus, comprehensive research is needed to inform locally relevant dietary guidelines that integrate health, sustainability, food systems, and sociocultural factors.

While it is anticipated that the PHD-NL is more sustainable than the DDG-NL, further research is required to validate its environmental impact. Additionally, assessing whether adherence to this pattern effectively contributes to achieving Sustainable Development Goals (SDGs) is necessary. Furthermore, it is necessary to conduct research on the nutritional adequacy of the PHD-NL for populations beyond adults aged 18 to 70 years, including children, older adults, and lactating women, who have differing nutritional requirements. Also, evaluating specific PHD-NL adaptations for vegetarian or vegan populations is required.

## 5. Conclusions

Overall, the PHD-NL diet is nutritionally adequate, although optimization of the diet to achieve better levels of calcium and vitamin D is necessary before it can be recommended to the Dutch population. Also, it is essential to include water recommendations in the PHD and expand research in hydration and environmental sustainability. Finally, the DDG coincides with several guidelines for PHD, indicating that current national guidelines in the Netherlands are compatible with sustainability as well as nutritional adequacy.

Finally, this work could be useful in supporting the updating and implementation of the Dutch Dietary Guidelines. Dietary advice has been given to the Dutch population since 1941, and the DDG has been updated periodically [66]. Some aspects to consider for upcoming DDG and public policies:

- DDG could evaluate the recommended amounts of dairy products, red meat, fish, legumes, nuts, and seeds to be consistent with the PHD;
- The DDG could emphasize consuming whole grain products and seeds beyond “brown” products to increase fiber consumption;
- Sugar intake must be addressed in the Dutch population, with some warnings and public policies (e.g., frontal package labeling, taxes, and marketing regulations) to prevent excessive consumption. The WHO recommends limiting the daily intake of added sugars in the diet.

## Figures and Tables

**Table 1:** Food groups and subgroups comparison from the Planetary Health Diet and the Dutch Dietary Guidelines.

Food Group PHD	Food Group DDG	PHD (Grams per Day)	DDG ^1^ (Grams per Day)	Numerical Difference (PHD-DDG)
Rice, wheat, corn, and other	Bread (brown bread)	232	205	27
Potatoes and cassava	Cereal products and potatoes	50	105	−55
All vegetables	Vegetables	300	250	50
All fruits	Fruits	200	200	0
Dairy	Milk and dairy products + cheese **	250	655	−405
Beef, lamb, and pork	Meat	14	23.8	−9.8
Chicken and other poultry	Chicken, poultry	29	23.8	5.19
Eggs	Eggs	13	23.8	−10.8
Fish	Fish	28	14.3	13.7
Legumes	Legumes	75	17.1	57.9
Nuts	Nuts (without salt)	50	15	35
Saturated	Fats and oils (spreadable and cooking fats)	51.8	40–65 Mean: 52.5	11.8 and −13.2 0.7
Unsaturated oils				
Added sugars	No equivalent group	31	0	31.0
No equivalent group	Drinks: water, coffee, or tea	0	1500	−1500

^1^ In the DDG, all amounts were the same for women and men, with exceptions for bread, whole cereals, and fats. ** The DDG includes cheese in a different group, and this was added to the dairy foods group to compare.

**Table 2:** Macronutrient contents and nutritional adequacy presented as % Dietary Reference Value (DRV) for Dutch males and females.

Nutrient	PHD-NL Nutritional Content	DDG-NL Nutritional Content	PHD-NL (%DRV)		DDG-NL (%DRV)	
			Men	Women	Men	Women
Energy (kcal)	1952	1968	-	-	-	-
Carbohydrates (g)	179	215	92	92	109	109
Carbohydrates (%En)	37	44	93	93	110	110
Mono- and disaccharides (g)	53	100	-	-	-	-
Sugars (g)	2.87	33	-	-	-	-
Polysaccharides (g)	126	115	-	-	-	-
Fiber (g)	39	30	97–111	130–156	74–85	99–119
Protein (g)	82	91	187–211	169–187	206–231	185–206
Protein (%En)	17	19	189–213	170–189	211–238	190–211
Vegetable protein (g)	51	32	-	-	-	
Animal protein (g)	31	59	-	-	-	
Total fat (g)	92	74	106–212	106–212	85–170	85–170
Total fat (%En)	42	34	105–210	105–210	85–170	85–170
Saturated fat (g)	21	19	-	-	-	
Saturated fat (%En)	9.9	8.9	99	99	89	89
Polyunsaturated fat (g)	29	24	-	-	-	
Polyunsaturated fat (%En)	13	11	108	108	92	92
Linoleic acid (g)	23	18	-	-	-	
Alpha linolenic acid (mg)	4.6	5.1	-	-	-	
Alpha linolenic acid (%En)	2.1	2.3	210	210	230	230
Eicosapentaenoic acid (mg)	120	67	-	-	-	
Docosahexaenoic acid (mg)	160	79	-	-	-	
Marine fatty acids (mg)	280	146	140	140	73	73
Monounsaturated fat (g)	35	24	-	-	-	
Unsaturated fat, cis (mg)	63	49	-	-	-	
Trans fat (g)	0.6	0.5	-	-	-	
Trans fat (%En)	0.3	0.2	30	30	20	20
Cholesterol (mg)	149	134	-	-	-	

Dietary Reference Values (DRVs) taken from [30,31]. A total of 35 g fiber is recommended for males aged 51–70 years and 40 g for 19–50 years. A total of 25 g fiber is recommended for females aged 51–70 years and 30 g for 19–50 years. DDG-NL: theoretical diet based on Dutch Dietary Guidelines. g: grams. mg: milligrams. PHD-NL: theoretical diet based on Planetary Health Diet from EAT-Lancet Commission. %DRV: Percentage Dietary Reference Value. %En: percentage from total energy.

**Table 3:** Micronutrient content of the PHD-NL and DDG-NL and their Dietary Reference Values (DRVs) for Dutch adults.

Nutrient	PHD-NL Nutritional Content	DDG-NL Nutritional Content	DRV	
			Men	Women
Vitamin A (µg) *	1740	2374	615	525
Vitamin B1 (mg) **	1.6	1.5	0.83	0.61
Vitamin B2 (mg)	1.7	2.4	1.3	1.3
Vitamin B3 (mg) **	22	20	15	11
Vitamin B6 (mg)	2.1	2.7	1.1	1.1
Vitamin B9 (µg)	455	411	200	200
Vitamin B12 (µg)	6.0	7.9	2	2
Vitamin C (mg)	130	165	60	50
Vitamin D (µg)	4.2	3.2	10	10
Vitamin E (mg)	19	16	13	11
Vitamin K (µg)	303	281	70	70
Calcium (mg) ***	794	1652	860	860
Copper (mg)	1.7	1.4	0.7	0.7
Iron (mg)	14	12	6	7
Iodine (µg)	209	175	150	150
Potassium (mg)	3517	4104	3500	3500
Magnesium (mg)	463	423	350	300
Sodium (mg)	1765	1739	2400	2400
Phosphorus (mg)	1524	1735	550	550
Selenium (µg)	93	55	70	70
Zinc (mg)	11	12	121	155

* Retinol activity equivalents for vitamin A. ** DRVs for vitamin B1 (Thiamine) and vitamin B2 (Niacin) were calculated for a diet of 2000 kcal/day for women and 2700 kcal/day for men. *** Calcium reference value averaged between 750 and 860 mg. Vitamins E, K, magnesium, phosphorus, and selenium were Adequate Intake values instead of the EAR. Sodium is the maximum intake recommended. Dietary Reference Values—DRVs (EAR/Average requirement and Adequate Intake) were taken from [31]. DDG-NL: theoretical diet based on Dutch Dietary Guidelines. g: grams. mg: milligrams. µg: micrograms. PHD-NL: theoretical diet based on Planetary Health Diet from EAT-Lancet Commission.

## Data Availability

Dutch National Food Consumption Survey 2012–2016 available at https://www.rivm.nl/bibliotheek/rapporten/2020-0083.pdf (accessed on 6 January 2023). Dutch food data composition can be consulted at https://www.rivm.nl/nederlands-voedingsstoffenbestand (accessed on 6 January 2023). More data can be consulted in Supplementary Materials or by writing to the corresponding author.

## Supplementary Materials

The following supporting information can be downloaded at https://www.mdpi.com/article/10.3390/nu16142219/s1, Supplementary Material S1: EAT-lancet guidelines; Supplementary Material S2: Dutch dietary guidelines; Supplementary Material S3: Nutrient criteria Dutch Dietary Guidelines DDG; Supplementary Material S4: Nutrient criteria PHD-NL diet and DDG-NL diet; Supplementary Material S5: Dutch nutrient recommendations; Supplementary Material S6: Products per food (sub)group PHDNL DDGNL Diets; Supplementary Material S7: PHD-NL DDG-NL Nutritional Content.
