# Supplementary material for: Planetary Health Diet Compared to Dutch Dietary Guidelines: Nutritional Content and Adequacy
Source: Nutrients. 2024 Jul 11;16(14):2219. doi: 10.3390/nu16142219 (PMC11280056; doi:10.3390/nu16142219)
Supplement: Supplementary file 1 [file nutrients-16-02219-s001.zip › Supplementary Material S1 EAT-lancet guidelines.pdf]

Supplementary Material S1 - EAT-lancet guidelines

Table S1 - Guidelines for the healthy reference diet based on the intake of 2500 kcal/day as developedby the EAT-Lancet Commission on healthy diets from sustainable food systems (16).

	Macronutrient intake (possible range) g/day	Caloric intake kcal /day
**Whole grains***		
Rice, wheat, corn and other ✝	232 (0-60 en%)	811
**Tubers or starchy vegetables**		
Potatoes and cassava	50 (0-100)	39
**Vegetables**		
All vegetables	300 (200-600)	
Dark green vegetables	100	23
Red and orange vegetables	100	30
Other vegetables	100	25
**Fruits**		
Fruits	200 (100-300)	126
**Dairy foods**		
Whole milk or derivate equivalents (e.g. cheese)	250 (0 – 500)	153
**Protein sources Ŧ**		
Beef and lamb	7 (0-14)	15
Pork	7 (0-14)	15
Chicken and other poultry	29 (0-58)	62
Eggs	13 (0-58)	62
Fish	28 (0-100)	40
Legumes		
Dry beans, lentils and peas*	50 (0 -100)	172
Soy foods	25 (0-50)	112
Peanuts	25 (0-75)	142
Tree nuts	25	149
**Added fats**		
Palm oil	6.8 (0-6.8)	60
Unsaturated oils (20% olive, soybean, rapeseed, sunflower and peanut oil)	40 (20-80)	354
Dairy fats (included in milk)	0	0
Lard or tallow ‡	5 (0-5)	36
**Added sugars**		
All sweeteners	31 (0-31)	120

* Wheat, rice, dry beans and lentils are dry, raw

✝ Mix and amount of grains can vary to maintain isocaloric intake

Ŧ Beef and lamb are exchangeable with pork and vice versa. Chicken and other poultry are exchangeable with eggs, fish or plant protein sources. Legumes, peanuts, tree nuts, seeds and soy are interchangeable.

* Some lard or tallow is optional in instances where pigs or cattle are consumed.
