# Supplementary material for: Planetary Health Diet Compared to Dutch Dietary Guidelines: Nutritional Content and Adequacy
Source: Nutrients. 2024 Jul 11;16(14):2219. doi: 10.3390/nu16142219 (PMC11280056; doi:10.3390/nu16142219)
Supplement: Supplementary file 1 [file nutrients-16-02219-s001.zip › Supplementary Material S2 Dutch dietary guidelines.pdf]

Supplementary Material S2. Dutch dietary guidelines

Table S2 - Dutch dietary guidelines for females aged 51 – 69, as developed by the Health council of the Netherlands. Portions and spoons are converted to grams by using Portie-Online).

	Intake
**Vegetables and fruits**	
Vegetables	250 g
Fruit	2 portions = 200 g
**Bread, grain/cereal products and potatoes**	
Brown/ whole grain sandwiches	3 - 4portions =<br>• 105 – 140 g bread, cornflakes or cereals
Whole grain products or potatoes	3 - 4 spoons =<br>• 210 - 280 g potatoes<br>• 135 - 180 g whole grain pasta<br>• 180 - 240 g whole grain rice
**Dairy, nuts, fish, legumes, meat and eggs**	
Fish/ Legumes/ meat/ eggs	1 portion =<br>• 100 g fish ‡<br>• 2-3 spoons legumes = 120 – 180 g<br>• 100 g meat*<br>• 2-3 eggs = 100 – 150 g
Unsalted nuts	15 g
Dairy	3 – 4 portions =<br>• 750 - 1000 g milk<br>• 450 – 600 ml dairy products
Cheese	40 g
**Spreading and cooking fats ☨**	
Spreadable fats and cooking fats	40 g
**Drinks Ŧ**	
Fluids	1.5 – 2 L
**Other products**	
Products that do not belong to one of the other food groups	15 en%

* Eat one serving of fish weekly, preferably oily fish

* Limit the consumption of red meat, particularly processed meat.

☨ Replace butter, hard margarine and cooking fats with soft margarine, liquid cooking fats, and vegetable oils.

Ŧ Drink three cups of tea daily, replace unfiltered coffee with filtered coffee, minimise the consumption of sugar-containing beverages and do not drink alcohol or drink no more than one glass daily.
