# Supplementary material for: Planetary Health Diet Compared to Dutch Dietary Guidelines: Nutritional Content and Adequacy
Source: Nutrients. 2024 Jul 11;16(14):2219. doi: 10.3390/nu16142219 (PMC11280056; doi:10.3390/nu16142219)
Supplement: Supplementary file 1 [file nutrients-16-02219-s001.zip › Supplementary Material S3 Nutrient criteria Dutch Dietary Guidelines DDG.pdf]

Supplementary Material S3 – Nutrient criteria Dutch Dietary Guidelines DDG (DHD-15)

Table S3 - Nutrient criteria to which products must comply, to be allocated to one of the food (sub-)groups of the DDG

	Nutrient criteria	Remarks
**Vegetables and fruits**		
Vegetables<br>• Unprocessed	No criteria	
Vegetables<br>• Processed	No drinks<br>Saturated fat: ≤0.5 g / 100 g<br>Trans fatty acids: ≤0.1 g / 100 g<br>Sodium: not added<br>Sugar: not added	
Fruit<br>• Unprocessed	No criteria	
Fruit<br>• Processed	No drinks<br>Saturated fat: ≤0.2 g / 100 g<br>Trans fatty acids: ≤0.1 g / 100 g<br>Sodium: not added<br>Sugar: not added	Max. 20g dried fruit without added sugar per day.
**Bread, grain/cereal products and potatoes**		
Bread and cereal products<br>• Brown/whole-grain sandwiches	Saturated fat: ≤1.0 g / 100 g<br>Trans fatty acids: ≤0.1 g / 100 g<br>Sodium: ≤450 mg / 100 g<br>Sugar: Total ≤8.5 g / 100 g<br>Fibre: ≥4.5 g / 100 g	
Bread and cereal products<br>• Dry products (e.g. crispbread)	Saturated fat: ≤1.0 g / 100 g<br>Trans fatty acids: ≤0.1 g / 100 g<br>Sodium: ≤450 mg / 100 g<br>Sugar: Total ≤8.5 g / 100 g<br>Fibre: ≥6.0 g / 100 g	
Bread and cereal products<br>• Breakfast cereals	Saturated fat: ≤2.0 g / 100 g<br>Trans fatty acids: ≤0.1 g / 100 g<br>Sodium: Not added<br>Sugar: Total ≤16 g / 100 g<br>Fibre: ≥8.0 g / 100 g	
Bread and cereal products<br>• Meal and bakery products	Saturated fat: -<br>Trans fatty acids: ≤0.1 g / 100 g<br>Sodium: Not added<br>Sugar: Not added<br>Fibre: ≥7.0 g / 100 g	
Bread and cereal products<br>• Bread and pizza mixes	Saturated fat: -<br>Trans fatty acids: ≤0.1 g /100 g<br>Sodium: ≤600 mg / 100 g<br>Sugar: Total ≤2.5 g / 100 g<br>Fibre: ≥7.0 g / 100 g	

Whole-grain products or potatoes	Saturated fat: ≤0.2 g / 100 g<br>Trans fatty acids: ≤0.1 g / 100 g

• Rice, pasta, couscous, millet, quinoa	Sodium: Not added<br>Sugar: Not added<br>Fibre: ≥2.1 g / 100 g	
Whole-grain products or potatoes<br>• Potatoes	Saturated fat: ≤0.1 g / 100 g<br>Trans fatty acids: ≤0.1 g / 100 g<br>Sodium: Not added<br>Sugar: Not added	
**Dairy, nuts, fish, legumes, meat and eggs**		
Fish/ legumes/ meat/ eggs<br>• Fish	No criteria	≥70% fish
Fish/ legumes/ meat/ eggs<br>• Legumes	Saturated fat: ≤0.8 g / 100 g<br>Trans fatty acids: ≤0.1 g / 100 g<br>Sodium: ≤200 mg / 100 g<br>Sugar: Not added	
Fish/ legumes/ meat/ eggs<br>• Meat unprocessed	Saturated fat: ≤5.0 g / 100 g<br>Trans fatty acids: Not added<br>Sodium: Not added<br>Sugar: Not added	
Fish/ legumes/ meat/ eggs<br>• Meat processed	Not allowed	
Fish/ legumes/ meat/ eggs<br>• Vegetarian products	Saturated fat: ≤2.5 g / 100 g<br>Trans fatty acids: ≤0.1 g / 100 g<br>Sodium: ≤450 mg / 100 g<br>Sugar: Not added<br>Iron: ≥0.8 mg / 100 g<br>Vitamin B12: ≥0.24 µg / 100 g and/or vitamin B1: ≥0.06 µg / 100 g<br>Protein: ≥ 20 en%	
Fish/ legumes/ meat/ eggs<br>• Eggs	No criteria	
Unsalted nuts<br>• Unprocessed	Saturated fat: -<br>Trans fatty acids: ≤0.1 g / 100 g<br>Sodium: Not added<br>Sugar: Not added	
Unsalted nuts<br>• Processed	Saturated fat: -<br>Trans fatty acids: ≤0.1 g / 100 g<br>Sodium: Not added<br>Sugar: Not added	
Dairy<br>• Milk and milk products	Saturated fat: ≤1.1 g / 100 g<br>Trans fatty acids: Not added<br>Sodium: Not added<br>Sugar: Not added or ≤6 g / 100 g	
Dairy<br>• Plant-protein drinks and desserts	Saturated fat: ≤1.1 g / 100 g<br>Trans fatty acids: Not added<br>Sodium: ≤60 mg / 100 g<br>Sugar: Total ≤6 g / 100 g<br>Calcium: ≥80 mg / 100 g	

	Vitamin B12: ≥ 0.24 µg / 100 g<br>Protein: ≥20 en%
Cheese	Saturated fat: ≤14 g / 100 g<br>Trans fatty acids: Not added<br>Sodium: ≤820 mg / 100 g<br>Sugar: Not added
Cheese<br>• Cheese replacer based on plant-based ingredients	Saturated fat: ≤14 g / 100 g<br>Trans fatty acids: Not added<br>Sodium: ≤820 mg / 100 g<br>Sugar: Not added<br>Calcium: ≥500 mg / 100 g<br>Vitamin B12: ≥ 0.24 µg / 100 g<br>Protein: ≥20 en%
**Spreading and cooking fats**	
Spreadable fats and cooking fats	Saturated fat: ≤30% of total fat<br>Trans fatty acids: ≤1.0 g / 100 g<br>Sodium: ≤160 mg / 100 g<br>Sugar: Not added
Drinks ₸	
Fluids<br>• Water, tea, coffee	Water, tea and filtered coffee without sugar
Fluids<br>• Soft drinks<br>• Fruit and vegetable juices<br>• Alcoholic drinks	Not allowed
**Other products**	
Products that do not belong to one of the other food groups	No criteria
