# Supplementary material for: Planetary Health Diet Compared to Dutch Dietary Guidelines: Nutritional Content and Adequacy
Source: Nutrients. 2024 Jul 11;16(14):2219. doi: 10.3390/nu16142219 (PMC11280056; doi:10.3390/nu16142219)
Supplement: Supplementary file 1 [file nutrients-16-02219-s001.zip › Supplementary Material S4 Nutrient criteria PHD-NL diet.pdf]

Supplementary Material S4 - Nutrient criteria PHD-NL diet

Table S4 - Nutrient criteria to which products must comply, to be allocated to one of the food (sub-)groups of the PHD-NL diet.

	Nutrient criteria
**Rice, wheat, corn and other**	
Bread	Fibre: ≥ 6.0 g/100 g<br>Sugar: Total ≤8.5 g / 100 g<br>Sodium: ≤450 mg / 100 g
Dry products such as crackers	Fibre: ≥ 6.0g/100 g<br>Sugar: Total ≤8.5 g / 100 g<br>Sodium: ≤450 mg / 100 g
Breakfast cereals	Fibre: ≥ 8.0 g/100 g<br>Sugar: Total ≤16 g / 100 g<br>Sodium: Not added
Meal and bakery products	Fibre: ≥11.0 g/100g<br>Sugar: Not added<br>Sodium: Not added
Rice, pasta, etc.	Fibre: ≥2.1 g/100g<br>Sugar: Not added<br>Sodium: Not added
**Potatoes and cassava**	
	Not processed †
**All vegetables**	
Dark green vegetables	Not processed †
Red and orange vegetables	Not processed †
Other vegetables	Not processed †
**Fruits**	
	Not processed †
**Whole milk or derivate equivalents (e.g. cheese)**	
Milk and milk products	Sodium: Not added<br>Sugar: Not added or total sugar ≤6 g / 100 g
Cheese	Sodium: ≤820 mg / 100 g<br>Sugar: Not added
**Protein sources**	
Beef, lamb and pork	Not processed †
Chicken and other poultry	Not processed †
Eggs	No criteria
Fish	Not processed †
Legumes	
Dry beans, lentils and peas	Sodium: ≤200 mg / 100 g<br>Sugar: Not added
Soy foods	Not processed †
Peanuts	Not processed †
Tree nuts	Not processed †
**Added fats**	
Palm oil	> 25% saturated fat
Unsaturated oils	≤ 25 % saturated fat

Lard or tallow	No criteria
**All sweeteners**	
	No criteria

† A product is considered not processed when it
