# Supplementary material for: Planetary Health Diet Compared to Dutch Dietary Guidelines: Nutritional Content and Adequacy
Source: Nutrients. 2024 Jul 11;16(14):2219. doi: 10.3390/nu16142219 (PMC11280056; doi:10.3390/nu16142219)
Supplement: Supplementary file 1 [file nutrients-16-02219-s001.zip › Supplementary Material S5 Dutch nutrient recommendations.pdf]

Supplementary Material S5 - Dutch nutrient recommendations

Table S5 - Dutch nutrient recommendations (DNRs) for macronutrients, 12 vitamins and 10 minerals. PRI: Population reference intake, AI: Adequate intake.

	Males	***Females***	***Upper limit***
**Macronutrients**			
Carbohydrate (en%)	40	40	
Fibre (g)	35 – 40	25 – 30	
Fat (en%)	20	20	40
Saturated fat (en%)			10
Polyunsaturated fat (en%)			12
Unsaturated fat, cis (en%)	8 - 38	8 - 38	
Unsaturated fat, trans (en%)			1
Linoleic acid (en%)	2	2	
Alpha linolenic acid (en%)	1	1	
Marine fatty acids (EPA + DHA) (g)	0.2	0.2	
Protein (en%)	8 – 9	9 – 10	25
**Vitamins**			
Vitamin A (mg)	PRI: 0.9	PRI: 0.7	3.0
Vitamin C (mg)	PRI: 75	PRI: 75	2000
Vitamin D (mg)	AI: 0.01	AI: 0.01	0.1
Vitamin E (mg)	PRI: 10	PRI: 8	300
Vitamin K (mg)	AI: 0.12	AI: 0.09	
Vitamin B1 (Thiamine) (mg)	PRI: 1.1	PRI: 1.1	
Vitamin B2 (Riboflavin) (mg)	PRI: 1.5	PRI: 1.1	
Vitamin B3 (Niacin) (mg)	PRI: 17	PRI: 13	900
Vitamin B6 (mg)	PRI: 1.5	PRI: 1.5	25
Vitamin B11 (Folic acid) (mg)	PRI: 0.3	PRI: 0.3	
Synthetic folate (mg)			1.0
Vitamin B12 (mg)	PRI: 0.0028	PRI: 0.0028	
**Minerals**			
Calcium (mg)	AI: 1000 – 1100	AI: 1000 – 1100	2500
Phosphorus (mg)	PRI: 600	PRI: 600	
Potassium (mg)	PRI: 3500	PRI: 3100	
Magnesium (mg)	PRI: 350	PRI: 280	
Iron (mg)	PRI: 9	PRI: 9	25
Zinc (mg)	PRI: 9	PRI: 7	25
Coper (mg)	PRI: 0.9	PRI: 0.9	5
Iodine (mg)	PRI: 0.15	PRI: 0.15	0.6
Selenium (mg)	PRI: 0.06	PRI: 0.05	0.3
Sodium (mg)			2400
