# Supplementary material for: Planetary Health Diet Compared to Dutch Dietary Guidelines: Nutritional Content and Adequacy
Source: Nutrients. 2024 Jul 11;16(14):2219. doi: 10.3390/nu16142219 (PMC11280056; doi:10.3390/nu16142219)
Supplement: Supplementary file 1 [file nutrients-16-02219-s001.zip › Supplementary Material S6 Products per food (sub)group PHDNL DDGNL Diets.pdf]

Supplementary Material S6 - Products per food (sub-)group

Table S6A - Products added to the PHD-NL diet.

	Products
**Whole grains**	
Rice, wheat, corn and other	• Bread rye dark<br>• Bread rye light<br>• Bread wholemeal average<br>• Bread wheatrye wholemeal<br>• Bread rye average<br>• Bread Blue Band Goede Start white bread<br>• Bread wholemeal average w pumpkin seeds<br>• Bread linseed<br>• Bread multigrain average w seeds<br>• Bread wholemeal w nuts<br>• Bread wholemeal average w sunflower seeds<br>• Bread Blue Band Goede Start light brown<br>• Bread wholemeal average w seeds<br>• Bread brown w seeds<br>• Bread wholemeal coarse<br>• Bread sourdough wholemeal<br>• Roll wholemeal soft<br>• Roll multigrain hard<br>• Roll multigrain soft<br>• Bread wholemeal fine<br>• Bread multigrain wholemeal Becel<br>• Bread wholemeal Brinta Vezelbruin<br>• Bread white gluten free Pan Carre Schar<br>• Bread multigrain gluten free Rustico<br>• Bread VollerKoren<br>• Bread white Brinta Vezelwit<br>• Bread wholemeal fine w seeds<br>• Bread wholemeal fine w sunflower seeds<br>• Bread wholemeal coarse w seeds<br>• Bread wholemeal coarse w sunflower seeds<br>• Bread wholemeal coarse w pumpkin seeds<br>• Bread Tijger wholemeal<br>• Bread corn w seeds<br>• Breakfast cereal Brinta<br>• Wheat germ<br>• Breakfast cereal porridge Bambix Zonnig Ontbijt licht volk<br>• Breakfast prod Albona 7--cereals--energy<br>• Breakfast cereal All--Bran flakes<br>• Muesli w fruit<br>• Breakfast cereal Weetabix original<br>• Crispbread averaged<br>• Crispbread light

	• Crispbread high fibre<br>• Crispbread wholemeal Cracottes<br>• Crispbread wholemeal<br>• Crispbread Sandwich Wasa<br>• Crackers VitaLU<br>• Crispbread Cracottes Vital<br>• Toast Melba other varieties<br>• Crispbread Oerknack Bolletje<br>• Crispbakes Dutch farmers cereals&seeds Bolletje<br>• Flour wheat wholemeal<br>• Wheat bran<br>• Barley whole grain raw<br>• Oat bran raw<br>• Rice brown boiled<br>• Pasta wholemeal boiled<br>• Quinoa cooked<br>• Bulgur wheat cooked
**Tubers or starchy vegetables**	
Potatoes and cassava	• Potato puree powder wo milkpowder wo fat<br>• Potatoes wo skins boiled average<br>• Potatoes slices/parts frozen unprepared<br>• Potato sweet boiled<br>• Potatoes boiled w skin average<br>• Potato product natural precooked chilled<br>• Potato product seasoned precooked chilled
**Vegetables**	
Dark green vegetables	• Endive raw<br>• Endive boiled<br>• Kale curly boiled<br>• Cucumber wo skin raw<br>• Cucumber boiled<br>• Sweet pepper green raw<br>• Sweet pepper green boiled<br>• Purslane boiled<br>• Leek boiled<br>• Turnip tops raw<br>• Cabbage Savoy boiled<br>• Lettuce butterhead raw<br>• Swiss chard leaf boiled<br>• Spinach raw<br>• Spinach boiled<br>• Brussel sprouts boiled<br>• Lettuce lambs raw<br>• Spinach tinned<br>• Spinach frozen boiled<br>• Purslane raw<br>• Broccoli boiled

- Broccoli raw
- Kale curly frozen boiled
- Kale curly raw
- Courgettes boiled
- Dandelion leaves raw
- Brussel sprouts frozen boiled
- Seaweed kelp raw
- Cucumber w skin raw
- Bitter gourd pods boiled
- Seaweed nori dried

Red and orange vegetables

- Beetroot raw
- Cabbage red raw
- Cabbage red boiled
- Tomatoes classic round raw
- Tomatoes classic round boiled
- Carrot raw average
- Carrot boiled average
- Carrot tinned
- Sweet pepper red raw
- Sweet pepper red boiled
- Beetroot boiled
- Pumpkin boiled
- Chili pepper raw
- Carrot bunched raw
- Carrot bunched boiled
- Carrot winter raw
- Carrot winter boiled
- Tomato vine raw
- Tomato cherry raw
- Tomato beef raw
- Tomato beef boiled
- Tomato average raw
- Tomato average boiled
- Pepper sweet orange raw
- Tomatoes dried in oil tin/glass
- Tomato sun--dried
- Tomato juice
- Juice tomato/vegetable
- Juice tomato/vegetable Appelsientje
- Juice tomato Appelsientje Zontomaat
- Tomato puree concentrated tinned
- Pepper red hot paste
- Pepper red hot fried paste
- Tomato sieved

Other vegetables

- Aubergine raw
- Aubergine boiled
- Celery boiled

- Cauliflower raw
- Cauliflower boiled
- Mushrooms chanterelle boiled
- Mushroom raw
- Mushroom boiled
- Cabbage Chinese boiled
- Celeriac boiled
- Swede boiled
- Kohlrabi boiled
- Cabbage oxheart raw
- Cabbage oxheart boiled
- Sweetcorn boiled
- Bean sprouts raw
- Bean sprouts boiled
- Onions raw
- Onions boiled
- Chicory raw
- Chicory boiled
- Cabbage white raw
- Cabbage white cooked
- Radish red/white raw
- Cress garden raw
- Vegetables mixture raw
- Asparagus white tinned
- Mushroom tinned
- Celery raw
- Celeriac raw
- Kohlrabi raw
- Leek raw
- Garlic raw
- Ginger root fresh
- Fennel raw
- Courgettes raw
- Asparagus white boiled
- Salsify boiled
- Fennel boiled
- Vegetable mixed Mexico frozen unprepared
- Bamboo shoots tinned
- Lettuce iceberg raw
- Horse--radish raw
- Vegetables average boiled
- Vegetables average raw
- Bami vegetables mixed boiled
- Lettuce average raw
- Lettuce red raw
- Rocket raw
- Onion Welsh raw

	• Onion Welsh boiled<br>• Sweet pepper yellow raw<br>• Sweet pepper yellow boiled<br>• Sweet pepper average raw<br>• Sweet pepper average boiled<br>• Sweetcorn tinned<br>• Vegetables for soup cooked<br>• Cabbage pak--choi cooked<br>• Parsnip raw<br>• Parsnip boiled<br>• Vegetables for stir fry Dutch cooked<br>• Vegetables for stir fry Italian cooked<br>• Vegetables for stir fry Oriental cooked<br>• Vegetables for stir fry Mushroom cooked<br>• Artichoke hearts canned<br>• Lettuce romaine raw<br>• Cabbage sauerkraut raw<br>• Cabbage sauerkraut cooked
**Fruits**	
Fruits	• Apple without skin average<br>• Strawberries<br>• Apricots w skin<br>• Pineapple<br>• Banana<br>• Blueberries<br>• Redcurrants<br>• Blackcurrants<br>• Bilberries<br>• Blackberries<br>• Lemon<br>• Cranberries fresh<br>• Grapes w skin average<br>• Raspberries<br>• Grapefruit<br>• Cherries<br>• Manderins<br>• Melon netted<br>• Pear wo skin<br>• Peach without skin<br>• Plums w skin<br>• Orange<br>• Fruit fresh citrus average<br>• Fruit fresh average excluding citrus<br>• Coconut meat fresh<br>• Plantain ripe raw<br>• Avocado<br>• Lime

- Mango
- Papaya
- Apple with skin average
- Pomegranate
- Figs fresh
- Kiwi fruit green
- Kaki / Sharon fruit
- Passion fruit
- Lychees
- Melon water
- Melon honeydew
- Nectarine
- Dates fresh
- Fruit fresh average including citrus
- Pear w skin
- Grapes black w skin
- Grapes white w skin
- Pineapple in own juice tinned
- Kiwi fruit yellow
- Melon cantaloupe
- Kiwi fruit average
- Raisins dried
- Apricots dried
- Dates dried
- Currants dried
- Prunes dried
- Fruit mixed dried
- Figs dried
- Abricots dried and soaked
- Prunes dried soaked in water
- Fruit mixed dried soaked in water
- Goji berries dried
- Mulberries raw
- Juice apple
- Juice redcurrant
- Juice grape
- Juice grapefruit
- Juice pear
- Juice lemon
- Juice pineapple
- Juice orange w pulp
- Juice orange with calcium Appelsientje
- Juice apple w vitamin C
- Juice orange Appelsientje Groeifruit
- Juice fruit Coolbest Vitaday tropical
- Juice fruit Hero Fruit&Co
- Juice fruit Vruchtoase multivit

- Juice orange freshly squeezed
- Juice multifruit mild w vit C
- Apple sauce wo sugar tinned
- Fruit snack Knijpfruit/Slurpfruit
- Olives green in brine tinned/glass
- Coconut milk
- Olives ripe in brine tinned/glass
- Olives average in brine tinned/glass
- Raisins soaked in water

Dairy foods

Whole milk or derivate equivalents (e.g. cheese)

- Cheese Gouda 48+ average
- Cheese 20+ Leidse w cumin/Fries clove
- Cheese Camembert 45+
- Cheese cottage
- Cheese cream 60+
- Cheese Saint Paulin/Port Salut
- Cheese cream soft Mon Chou
- Cheese Gruyere
- Cheese Emmenthaler
- Cheese Cheddar
- Cheese cream soft Boursin
- Cheese 48+ less salt average
- Cheese spread 60+ Kiri
- Cheese cream soft Paturain
- Cheese 30+ average
- Cheese Brie 60+
- Cheese goat fresh
- Cheese 20+
- Cheese 50+
- Cheese Dutch in Swiss--style 45+
- Cheese 45+
- Cheese Mozzarella made from cow's milk
- Cheese spread Eru Balans
- Cheese spread Eru kids
- Cheese goat hard
- Dairy spread plain/herbs light
- Cheese Gouda 48+ age 4--8 weeks
- Cheese Gouda 48+ age 8 wk--4 mths
- Cheese Gouda 48+ age 4--7 mths
- Mascarpone cheese
- Cheese spread 45+
- Cheese 30+ age 4--8 weeks
- Cheese 30+ age 8 wk--4 mths
- Cheese 30+ age 4--7 mths
- Cheese 30+ age 10--12 mths
- Cheeseprod w veg fat Kees extra gerijpt
- Cheese 30+ less salt age 4--8 weeks

- Cheese 30+ less salt age 8 wk--4 mths
- Cheese 30+ less salt age 4--7 mths
- Cheese 48+ less salt age 4--8 weeks
- Cheese 48+ less salt age 8 wk--4 mths
- Cheese 48+ less salt age 4--7 mths
- Cheese cream with herbs 25--30 g fat
- Cheese Ricotta
- Yoghurt l fat w fruit/van w sw Optimel
- Milk raw
- Coffee creamer w pufa Becel
- Yoghurt full fat
- Milk whole
- Coffee creamer full fat
- Coffee creamer half fat
- Milk semi--skimmed
- Buttermilk curds fresh
- Porridge oatmeal
- Buttermilk
- Coffee creamer low fat
- Coffee creamer 20% fat
- Milk skimmed
- Cream whipping
- Yoghurt Bulgarian whole milk
- Yoghurt low fat
- Fromage frais low fat
- Fromage frais half fat
- Fromage frais full fat
- Cream sour
- Yoghurt Bulgarian low fat
- Yoghurt half fat
- Creme fraiche
- Yoghurt drink w sweeteners Optimel
- Coffee creamer Balance
- Milk goats-- full fat
- Fromage frais low fat w fruit w sw
- Yoghurt drink w sweetener
- Yoghurt drink Fristi with sweetners
- Creme fraiche half fat
- Cream cooking
- Cream cooking light
- Cream type prod Blue Band Finesse
- Yoghurt drink Topvit w sweetner
- Yoghurt Greek full fat
- Yoghurt 0% fat w fruit Activia
- Dairy spread plain/herbs
- Yoghurt drink Actimel 0% fat natural
- Milkdrink skimmed milk Becel pro--activ

- Yoghurt low fat with fruit w sweetener
- Yoghurt full fat natural Activia
- Milk chocolate--flavoured w sw Optimel
- Yoghurt Turkish 4% fat
- Yoghurt Turkish 10% fat
- Cream whipped low fat w sweetener canned
- Yogurt low fat Activia
- Milk semi--skimmed lactose free
- Yoghurt drink w sweeteners enriched w calcium
- Yoghurt drink Optimel Puur
- Milk skimmed dried
- Milk whole dried

Protein sources

Beef, lamb and pork

- Bacon fat raw
- Beef rump steak raw
- Beef tenderloin steak raw
- Beef frying steak raw
- Minced beef raw
- Beef rib raw
- Beef stewing meat raw
- Beef sirloin rolled raw
- Beef roast raw
- Beef shank raw
- Beef rib steak raw
- Beef streaked/marbled raw
- Beef stewing steak raw
- Beef steak tartare raw
- Pork fillet raw
- Pork fricandeau part of leg raw
- Pork chop raw
- Minced pork raw
- Pork tenderloin raw
- Pork gammon steak raw
- Pork loin chop raw
- Pork shoulder chop raw
- Liver pork raw
- Pork spare rib raw
- Pork tenderloin medaillon raw
- Pork shoulder raw
- Bacon rasher raw
- Minced beef/pork raw
- Hamburger raw
- Veal olive raw
- Lamb leg raw
- Minced lamb raw
- Lamb chop raw
- Lamb shoulder raw

- Pork 5--14% fat raw
- Veal <5% fat raw average
- Lamb >10 g fat raw average
- Pork rib chop raw
- Pork schnitzel not breaded raw
- Kidney ox raw
- Minced meat raw average
- Meat average raw excl liver
- Beef average raw
- Beef for stewing averaged raw
- Beef olives raw
- Minced meat (50:50 pork beef) w veg raw
- Minced veal raw
- Veal frying steak raw
- Veal rib steak raw
- Veal stewing steak raw
- Veal stewing meat raw
- Pork stewing meat raw
- Bacon lean smoked raw
- Pork shoarma seasoning raw
- Pork sparerib raw
- Beef ribeye steak raw
- Beef <5% fat raw average

Chicken and other poultry

- Duck whole raw
- Chicken w skin raw
- Pheasant whole raw
- Turkey raw
- Liver chicken raw
- Chicken wo skin raw
- Chicken for soup w skin raw
- Chicken fillet raw
- Chicken rolled raw
- Turkey fillet raw
- Chicken drumstick w skin raw
- Mince chicken raw

Eggs

- Egg whole chicken average boiled
- Egg yolk chicken boiled
- Egg white chicken raw

Fish

- Mussels boiled
- Fish lean 0--5 g fat raw
- Kipper smoked
- Shrimps Dutch peeled boiled
- Crab in water tinned
- Lobster boiled
- Sardines/pilchards in oil tinned
- Salmon tinned
- Eel smoked

- Mackerel steamed
- Cod boiled
- Plaice boiled
- Salmon smoked
- Halibut smoked
- Mackerel fillet smoked
- Salmon farmed raw
- Anchovy in oil canned
- Tuna in oil tinned
- Tuna in water tinned
- Mackerel prepared in microwave oven
- Mackerel in oil tinned
- Salmon farmed prep in microwave oven
- Trout prepared in microwave oven
- Rainbow trout prepared in microwave oven
- Dab/lemon dab prep in microwave oven
- Sole prepared in microwave oven
- Ocean perch prepared in microwave oven
- Eel prepared in microwave oven
- Spawn/hard roe coloured
- Squid prepared in microwave oven
- Tuna raw
- Pangasius prep in microwave oven
- Tuna prepared without fat
- Tilapia prepared without fat
- Anchovy raw
- Anchovy prepared without fat
- Sardines grilled
- Pollock Alaska steamed
- Prawns cooked
- Surimi

Legumes

Dry beans, lentils and peas

- Pea garden super fine tinned
- Beans runner tinned
- Beans French tinned
- Beans broad tinned
- Peas marrowfat canned
- Beans brown canned
- Beans French boiled
- Peas frozen boiled
- Beans French frozen boiled
- Beans runner boiled
- Beans broad boiled
- Peas fresh boiled
- Mange--tout boiled
- Peas marrowfat boiled
- Lentils green and brown boiled

	• Beans soya boiled
	• Peas chick boiled
	• Beans long yard Kouseband boiled
	• Splitpeas yellow boiled
	• Beans white canned
	• Beans mung boiled
	• Beans kidney red canned
	• Chickpeas canned
	• Beans black eyed canned
	• Beans brown boiled
	• Lentils brown canned
	• Peas marrowfat young canned
	• Beans kidney red boiled
	• Lentils red boiled
	• Beans white boiled
Soy foods	• Tahoe soya curd
	• Flour soya full fat
	• Quorn pieces unprepared
	• Drink soya Light Alpro
	• Drink soya unsweetened Alpro
	• Drink soya unsweetened Milbona/Albert Heijn
	• Drink almond unsweetened Alpro
	• Tempeh fermented soya beans
	• Miso soya paste
Peanuts	• Peanuts unsalted
	• Peanuts dry roasted
Tree nuts	• Almonds blanched unsalted
	• Cashew nuts unsalted
	• Hazelnuts unsalted
	• Chestnuts raw
	• Brazil nuts unsalted
	• Mixed nuts and raisins
	• Walnuts unsalted
	• Nuts mixed unsalted
	• Sesame seeds
	• Linseeds
	• Sunflower seeds
	• Pecan nuts unroasted unsalted
	• Pistachio nuts salted
	• Pine nuts
	• Poppy seeds
	• Pumpkin seeds
	• Nuts macadamia unsalted
	• Hemp seed
	• Chia seeds dried
**Added fats**	
Saturated fat	• Butter unsalted

- Butter salted
- Butter product half fat
- Low fat marg prod Blue Band Goede Start
- Low fat margarine 40% fat <17 g sat
- Low fat marg prod 20--25% fat <10 g sat
- Margarine 80% fat > 24 g saturates
- Margarine product 70% fat >17 g sat
- Cooking fat solid 97% fat >17 g sat
- Frying fat solid
- Cooking fat solid 80% fat >17 g sat
- Butter product 25% fat
- Low fat margarine prod Blue Band Idee
- Margarine 80% fat > 24 g sat unsalted
- Cooking fat sol 97% fat>17g sat unsalted
- Low fat margarine prod AH chol verlagend
- Low fat margarine prod C1000 Goed begin
- Low fat marg prod Dieetella Double Activ
- Low fat margarine product Gezonde Start
- Butter product melange unsalted Campina Botergoud
- Margarine product 60% fat >17 g sat
- Herb butter
- Oil coconut
- Low fat margarine 40% fat <17g sat w vit E
- Margarine product Jumbo bewust
- Butter product melange salted

Unsaturated fat

- Oil peanut
- Oil soy
- Oil sunflower seed
- Oil olive
- Oil Becel
- Oil safflower
- Oil corn
- Low fat margarine product tub Becel Ligh
- Margarine product tub Becel Dieet
- Low fat spread Becel pro--activ
- Low fat margarine prod 35% fat <10 g sat
- Margarine 80% fat < 24 g saturates
- Margarine product 70% fat <17 g sat
- Cooking fat liquid 97% fat <17 g sat
- Frying fat liquid
- Margarine product 60% fat <17 g sat
- Margarine liq 80% fat <17 g saturates
- Low fat margarine Gouda's Glorie Lichtste Pondje
- Low fat margarine prod Becel Omega3 Plus
- Oil wok
- Frying fat horeca

	• Margarine liq 80% fat < 17g sat unsalted<br>• Low fat margarine prod Bewust light<br>• Cooking fat liq 97%fat <17g sat unsalted<br>• Low fat marg 40% fat <17 g sat unsalted<br>• Margarineprod AlbertHeijn Bewust Balans<br>• Margarine product liquid light unsalted<br>• Margarine product liquid light<br>• Low fat marg prod Becel calorie light<br>• Low fat spread AH omega--3<br>• Low fat spead Becel pro,activ light<br>• Oil linseed<br>• Margarine product tub Jumbo diet<br>• Low fat margarine product Goed Begin Albert Heijn/Jumbo<br>• Margarine product Vita d'Or Bewust<br>• Low fat margarine product Vita d'Or Goed begin<br>• Oil rice bran<br>• Frying oil<br>• Margarine liquid 80% fat Blue Band<br>• Margarine liquid 80% fat Becel Olijf<br>• Margarine liquid 80% fat Vita d'Or Bewust<br>• Cooking fat liq Vita d'Or vlees&jus<br>• Low fat margarine product Vita d'Or Bewust light<br>• Oil sesame<br>• Oil rapeseed<br>• Oil vegetable average
Lard or tallow	• Lard<br>• Beef fat
**Added sugars**	
All sweeteners	• Sugar castor brown<br>• Sugar castor white<br>• Sugar granulated<br>• Syrup Keukenstroop<br>• Syrup sugar<br>• Syrup apple<br>• Honey<br>• Sweets boiled<br>• Cocoa powder sweetened<br>• Preparation glucose powder Dextro--M<br>• Sugar demerara<br>• Soft drink cola w caffeine<br>• Soft drink wo caffeine<br>• Juice orange pasteurized<br>• Juice drink<br>• Whey drink Rivella wo sugar<br>• Fruit drink concentrate Roosvicee Origin<br>• Fruit drink conc Roosvicee Low Cal

- Whey drink Taksi w sugar
- Fruit juice dk minimal 2 fruits
- Juice drink light
- Soft drink light wo caffeine
- Cola light soft drink w caffeine
- Fruit juice concentrated
- Fruit drink concentrate Karvan Cevitam
- Fruit juice drink Roosvicee Multivit
- Fruit drink conc Roosvicee Multivit
- Mineral water Sourcy
- Sports drink AA Isotone
- Sports drink AA High Energy
- Mineral water Bar le Duc
- Breakfast drink HeroFruitontbijt p 100ml
- Ice tea
- Ice tea light
- Ice tea with sugar and sweetener
- Juice drink Dubbelfrisss
- Juice drink Tintelfruit w vit C
- Juice drink Dubbelfrisss light
- Juice drink Tintelfruit light
- Juice drink Spa&Fruit still
- Juice drink Spa&Fruit light still
- Mineral water w sweetener
- Juice fruit Multi Vitamientje oranje vr
- Fruit drink Multi Vitamientje bosvr
- Juice fruit Coolbest Vitaday original
- Fruit juice dk Coolbest skinny orange
- Fruit drink 'portiefruit'
- Sports drink Extran Energy
- Sports drink Extran Hydro
- Fruit drink conc w sugar and sweetners 40--45g CHO
- Fruit drink concentrate light
- Fruit drink conc can Albert Heijn
- Rice drink w calcium and vitamines
- Juice drink Wicky
- Soft drink w sugar & sweetener 2--<5 g KH
- Juice drink w sugar & sw
- Juice drink multivitamin Capri--Sun
- Fruit drink conc diluted average
- Fruit drink conc w sugar diluted AH
- Fruit drink conc diluted 1 to 4
- Fruit drink conc diluted 1 to 7
- Sport drink River Powerdrink/Freeway
- Coffee Cappuccino freshly made
- Coffee cappuccino instant prepared

- Coffee cappuccino instant powder
- Energy drink Golden Power/Bullit/Freeway
- Fruit juice dk Roosvicee Original/frdr
- Fruit drink conc Roosvicee diluted av
- Fruit juice drink ACE average
- Fruit drink conc light diluted average
- Fruitdrink conc KarvanCevitam diluted av
- Spread speculaas flavoured
- Fruit juice drink 12 fruits nectar light
- Lemonade fruit Wicky zero
- Juice multifruit
- Fruit juice drink ACE Aldi/Kruidvat
- Juice fruit Solevita multivit 12--fruits
- Fruit juice drink Fruity King tropical
- Fruit juice concentrated prepared
- Fruit juice dk Surango multivit light
- Fruit juice dk minimal 2 fruits w vit C
- Fruit juice drink diary Wicky fruitzacht
- Fruit juice dk Solevita multivit 12 fru
- Fruit juice dk Roosvicee Multivit light
- Fruit juice dk ACE Fruxano/Super/Tasting
- Soft drink w sugar sw&caffeine 2--<5 carb
- Fruit drink conc w sugar & sw diluted
- Fruit drink Roosvicee 50/50
- Sports drink Aquarius
- Coffee w sugar and milk vending machine
- Coffee w milk vending machine
- Lemonade fruit Sisi no bubbles Action
- Softdrink w sugar sw&caffeine 5--<8g carb
- Mix rice/Chines noodles unprepared
- Energy drink Red Bull
- Energy drink Red Bull sugarfree
- Energy drink Mixxed up
- Fruit drink Linessa Vital&Active light
- Ice tea with less sugar
- Hot chocolate from vending machine
- Fruitdrink conc w sugar & sw 10--15 g CHO
- Fruitdrink conc w sug&sw diluted Tasting
- Whey drink Taksi w sweetener
- Sport drink Freeway Sportivo
- Coffee wiener melange instant powder
- Coffee wiener melange instant prepared
- Juice drink Spa&Fruit sparkling
- Juice Drink Ocean Spray
- Fruit drink concentrated Vruchtenmix
- Juice drink w sugar & sw Wicky
- Fruit juice drink apple nectar

- Fruit drink conc w sugars and sweeteners
- Fruit drink conc w 45--50 mg vit C
- Fruit drink conc w vit
- Vitaminwater
- Whey drink
- Fruit drink concentrated Vruchtenmix diluted
- Juice multifruit-- with vitamin E Healthy People
- Fruit juice drink juice and water
- Rice drink
- Fruit drink conc w sugar and sweeteners diluted
- Fruit drink conc w vit diluted
- Fruit drink concentrate with sweeteners Karvan Cevitam
- Fruit drink conc with sweetener Karvan Cevitam diluted
- Biscuit fortified Liga Baby 6--12 mnth
- Biscuit fort Liga 12--36 mnth
- Cake Dutch spiced Ontbijtkoek
- Almond filled pastry
- Apple pie Dutch w shortbread w marg
- Biscuit sweet
- Cake wo butter
- Cake sponge Dutch Eierkoek
- Gateau w whipped cream
- Gateau with butter--cream filling
- Biscuit average
- Coconut macaroons
- Biscuit sponge fingers
- Biscuit Dutch shortbread sprits
- Biscuit brown/wholemeal
- Chewing gum
- Chewing gum wo sugar
- Cream slice Dutch Tompouce
- Doughnut Dutch style w currants raisins
- Biscuit chocolate coated
- Biscuit shortbread Bastogne
- Sweets marshmellow type Spekkie
- Flan w fruit filling
- Candybar Mars
- Flan filled w rice pudding
- Liquorice Dutch type salted
- Liquorice Dutch type double salted
- Liquorice Dutch type sweet
- Liquorice Stophoest
- M&M's chocolate
- Candybar Milky Way
- Candybar Bounty

- Candybar Snickers
- Sauce fruit for pudding
- Sauce chocolate for pudding
- Sauce tomato based shashlik
- Cocoa product powder Ovomaltine
- Popcorn popped wo oil natural
- Meringue cake Bokkenpootje
- Coconut flavoured cookies
- Biscuit Dutch Krakeling
- Biscuit muesli
- Cassava cake Bojo Surinam
- Waffle syrup average
- Flan filling tinned
- Marsh mallows
- Liquorice allsorts
- Wine gum/ fruit gum
- Cake butter Dutch Boterkoek
- Apple strudel
- Cheesecake made w fromage frais
- Shortbread
- Biscuit oatmeal
- Candybar Twix
- Gateau fatless sponge w fruit & cream
- Biscuit spiced Speculaas w almond paste
- Sweets fruit pectinbased
- Biscuit fortifd w currants LigaEvergreen
- Cake Dutch spiced Ontbijtkoek wholemeal
- Sports food energy bar Isostar
- Biscuit fortified Liga Baby 4--6 mnth
- Biscuit fortified Liga Haverkick
- Biscuit gluten free Glutafin
- Biscuit fortified Liga Evergr assortment
- Turkish Delight
- Baklava nut--honey cake
- Liquorice Dutch type average
- Cake Dutch spiced Ontbijtkoek w ginger
- Syrup from tinned fruit
- Berliner pastry
- Biscuit with chocolate
- Butter cream
- Doughnut plain
- Ice lolly
- Eclair w whipped cream filling
- Meringue w butter--cream
- Biscuits sugar free
- Flan fruit and crumble topping
- Cheesecake made w cream cheese

- Biscuit Jaffa cakes/Cake PiM's
- Rice cakes puffed with salt
- Fondant cream
- Chocolates filled/Belgium chocolate
- Muesli bar w chocolate
- Eclair without filling
- Bun with vanilla custard
- Biscuit fortified Liga Milkbreak milkbiscuit
- Cake made w butter
- Biscuits assorted w butter
- Tart fruit w shortbread base
- Biscuit Cafe noir
- **f**Biscuit fortified Liga Continue vit/min
- Biscuit filled
- Biscuit w chocolate layer Scholiertje
- Rice cakes puffed with chocolate
- Biscuit fruit
- Cake Dutch spiced Ontbijtkoek w raisin
- Cereal bar Hero B'tween
- Cereal bar w milk Kellogg's
- Muesli bar
- Ice lolly Festini
- Ice cream dairy w chocolate coating
- Ice cream dairy w fruitcoating
- Boiled sweets sugar free
- Cake Dutch spiced Ontbijtkoek less sugar
- Rice cakes puffed with caramel
- Tiramisu
- After eight chocolate mints
- Chocolate milk w raisins
- Chocolate dark w nuts
- Chocolate milk w puffed rice
- Toffee w chocolate
- Nougat w chocolate
- Liquorice w peppermint
- Liquorice w sal ammoniac powder
- Nougat
- Popcorn popped wo oil sweet
- Cake marble--
- Cake apple--
- Cake raisins--
- Eclair filled w banana and whipped cream
- Chocolate eclair
- Chocolate pastry w whipped cream
- Cake Dutch spiced Ontbijtkoek w nuts
- Cake Dutchspiced Ontbijtkoek w rockcandy
- Spiced cake Indonesian-- Spekkoek

- Croissant chocolate--
- Tarts filled w jam
- Cup cake iced
- Cake Dutch spiced Lekkere Start
- Chocolate chip cookie
- Biscuit children's average
- Biscuit fortified Provita milkbiscuit
- Biscuit fortified Bridge fruit and grain
- Biscuit Dutch shortbread sprits w choc
- Teacakes chocolate coated marshmellow
- Biscuit Dutch Kletskop
- Cake wrapped in marzipan and chocolate
- Biscuit fortified Bridge milkbiscuit
- Ice cream stracciatella--
- Cake Dutch w icing & cream 'Oranjekoek'
- Wafer galette
- Spice biscuit sprinkles Bolletje
- Wafer w milk & hazelnts Knoppers
- Cake with nuts
- Fritter banana
- Gateau fatless sponge w marzipan
- Nuts cake
- Pudding airy
- Maltesers
- Liquorice sugarfree
- Wine gum w liquorice
- Biscuit fortified LU Time Out
- Apple turnover
- Flan w custard and crumble topping
- Cake Dutch Ontbijtkoek w nuts&fruit
- Flan w custard and fruit
- Cake made w butter and apple
- Biscuit peanut
- Candybar KitKat
- Biscuit w nuts and chocolate
- Biscuits w nuts
- Appel pie Dutch w shortbread w butter
- Waffle Luikse
- Waffle soft--/sugar--/flash--
- Biscuit spiced small Kruidnoten w dark choc
- Biscuit spiced small Kruidnoten w milk choc
- Biscuit Dutch Amaretti Bitterkoekjes
- Cake chocolate made wo butter
- Biscuit Dutch Frou frou
- Biscuit w dried fruit & yoghurt Yofruit
- Biscuit spiced small Kruidnoten w white choc
- Biscuit fortified Evergreen crunchy

- Doughnut iced
- Waffle Luikse w chocolate
- Almond paste filled tarts w butter
- Almond paste filled tarts wo butter
- Biscuit spiced Speculaas w butter
- Biscuit spiced Speculaas wo butter
- Biscuit brown/digestive w chocolate
- Flan apple and crumble topping
- Flan hard shell w custard cream
- Flan sponge w custard cream
- Waffle syrup w/o butter
- Waffle syrup w butter
- Chocolate bar filled Kinder
- Cake chocolate made w butter
- Pie apple--nuts
- Biscuit syrup w chocolate
- Candybar Lion
- Saus caramel for pudding
- Biscuit Dutch Jodekoek
- Waffle penny--
- Cake Indonesian
- Biscuit digestive
- Apple turnover w puff pastry w butter
- Sweets cream hard
- Puff pastry w butter baked
- Doughnut Dutch style plain
- Brownie without filling
- Biscuit Dutch Taai--taai
- Aniseed comfits crushed/pink&white/blue&white
- Mentos cheewy dragee
- Filopastry unprepared
- Sorbet
- Biscuit fortified AH/Jumbo/Aldi
- Biscuit fortified Liga Belvita ontbijtbiscuits
- Frogs/mice fondant with chocolate
- Cake Dutch spiced ontbijtkoek w chocolate
- Biscuit spiced small
- Icecream with caramel and nuts
- Cocoa powder
- Chocolate milk
- Chocolate dark
- Chocolate flakes milk
- Chocolate butter
- Chocolate flakes dark
- Spread chocolate hazelnut
- Coloured confetti fruit--flavoured
- Spread chocolate dark

- Jam
- Peppermint
- Jam rose hip
- Toffees
- Fruit drink concentrate undiluted
- Jam reduced sugar
- Chocolate bar milk w nuts
- Chocolate liqueurs
- Chocolate bar milk without sugar
- Marzipan
- Chocolate confetti averaged
- Milk chocolate--flavoured semi--skimmed
- Chocolate confetti milk
- Chocolate confetti dark
- Spread chocolate milk
- Milk chocolate--flavoured w sweetener w inulin
- Chocolate white
- Cocoa product sweetened Nesquik Hot Choc
- Chocolate confetti white
- Drinking chocolate w s--sk milk
- Drinking chocolate w s--sk milk and Nesquik
- Cocoa powder sweetened Nesquik
- Chocolate confetti mix white and dark
- Spread duo w chocolate
- Spread duo wo chocolate
- Sweets jelly/gums/foam
- Wine gums with foam layer
- Sweet fruity chew
- Sugar light
- Meringue
- Chocolate spread white
- Syrup maple
- Syrup apple enriched w iron
- Golden syrup pourable
- Fudge/caramel soft
- Chocolate flakes mix dark and white
- Chocolate confetti extra dark
- Marmalade
- Chocolate flakes white
- Syrup ginger
- Solid foamed candy 'schuimpje'
- Syrup apple--pear
- Liquorice Dutch type sweet with foam
- Sugar powdered
- Chocolate filled with caramel Rolo
- Icing (sugar)
- Chocolate extra dark

Supplementary Material S6 - Products per food (sub-)group

Table S6B - Products added to the DDG-NL diet.

Products
**Vegetables and fruits**

Vegetables

- Bean sprouts raw
- Bean sprouts boiled
- Endive raw
- Endive boiled
- Artichoke hearts canned
- Asparagus white boiled
- Asparagus white tinned
- Aubergine raw
- Aubergine boiled
- Bamboo shoots tinned
- Beetroot raw
- Beetroot boiled
- Celery boiled
- Celery raw
- Broccoli boiled
- Broccoli raw
- Mushrooms chanterelle boiled
- Mushroom raw
- Mushroom boiled
- Mushroom tinned
- Courgettes boiled
- Courgettes raw
- Ginger root fresh
- Vegetables average boiled
- Bami vegetables mixed boiled
- Vegetable mixed Mexico frozen unprepared
- Vegetables for stir fry Mushroom cooked
- Vegetables for stir fry Dutch cooked
- Vegetables for stir fry Italian cooked
- Vegetables for stir fry Oriental cooked
- Capers
- Garlic raw
- Celeriac boiled
- Celeriac raw
- Cucumber wo skin raw
- Cucumber boiled
- Cucumber w skin raw
- Vegetables average raw
- Cauliflower raw
- Cauliflower boiled
- Kale curly boiled
- Kale curly frozen boiled
- Kale curly raw
- Cabbage Chinese boiled
- Cabbage red raw

- Cabbage red boiled
- Cabbage Savoy boiled
- Cabbage oxheart raw
- Cabbage oxheart boiled
- Cabbage white raw
- Cabbage white cooked
- Cabbage sauerkraut raw
- Swede boiled
- Kohlrabi boiled
- Kohlrabi raw
- Beans long yard Kouseband boiled
- Sweetcorn boiled
- Sweetcorn tinned
- Horse--radish raw
- Cabbage pak--choi cooked
- Sweet pepper average raw
- Sweet pepper average boiled
- Sweet pepper yellow raw
- Sweet pepper yellow boiled
- Sweet pepper green raw
- Sweet pepper green boiled
- Pepper sweet orange raw
- Sweet pepper red raw
- Sweet pepper red boiled
- Parsnip raw
- Parsnip boiled
- Pumpkin boiled
- Purslane boiled
- Purslane raw
- Leek boiled
- Leek raw
- Turnip tops raw
- Radish red/white raw
- Vegetables mixture raw
- Salsify boiled
- Onions raw
- Onions boiled
- Lettuce average raw
- Lettuce red raw
- Lettuce iceberg raw
- Lettuce butterhead raw
- Rocket raw
- Dandelion leaves raw
- Lettuce romaine raw
- Lettuce lambs raw
- Swiss chard leaf boiled
- Vegetables for soup cooked

- Bitter gourd pods boiled
- Chili pepper raw
- Spinach raw
- Spinach boiled
- Spinach tinned
- Spinach frozen boiled
- Brussel sprouts boiled
- Brussel sprouts frozen boiled
- Tomatoes tinned
- Tomato average raw
- Tomato average boiled
- Tomato sun--dried
- Tomatoes classic round raw
- Tomatoes classic round boiled
- Tomato cherry raw
- Tomato vine raw
- Tomato beef raw
- Tomato beef boiled
- Tomato sieved
- Cress garden raw
- Onion Welsh raw
- Onion Welsh boiled
- Fennel raw
- Fennel boiled
- Chicory raw
- Chicory boiled
- Carrot raw average
- Carrot boiled average
- Carrot tinned
- Carrot winter raw
- Carrot winter boiled
- Carrot bunched raw
- Carrot bunched boiled

Fruit

- Strawberries
- Apricots w skin
- Pineapple
- Pineapple in own juice tinned
- Apple without skin average
- Apple with skin average
- Apple sauce tinned
- Apple sauce wo sugar tinned
- Avocado
- Banana
- Plantain ripe raw
- Blueberries
- Bilberries
- Redcurrants

- Blackcurrants
- Blackberries
- Lemon
- Cranberries fresh
- Dates fresh
- Grapes w skin average
- Grapes black w skin
- Grapes white w skin
- Raspberries
- Fruit fresh average including citrus
- Fruit fresh average excluding citrus
- Pomegranate
- Grapefruit
- Kaki / Sharon fruit
- Cherries
- Kiwi fruit average
- Kiwi fruit yellow
- Kiwi fruit green
- Coconut meat fresh
- Lime
- Lychees
- Manderins
- Mango
- Melon netted
- Melon honeydew
- Melon cantaloupe
- Melon water
- Fruit fresh citrus average
- Mulberries raw
- Nectarine
- Papaya
- Passion fruit
- Pear wo skin
- Pear w skin
- Peach without skin
- Plums w skin
- Orange
- Figs fresh
- Apricots dried
- Abricots dried and soaked
- Raisins soaked in water
- Goji berries dried
- Dates dried
- Currants dried
- Raisins dried
- Prunes dried
- Prunes dried soaked in water

	• Fruit mixed dried<br>• Fruit mixed dried soaked in water<br>• Figs dried
**Bread, grain/cereal products and potatoes**	
Brown/whole-grain sandwiches	• Bread brown wheat<br>• Bread brown/wholemeal average<br>• Bread linseed<br>• Bread brown wheat low sodium<br>• Bread multigrain average w seeds<br>• Bread Blue Band Goede Start light brown<br>• Bread wholemeal coarse<br>• Bread wholemeal Brinta Vezelbruin<br>• Bread wheat Vikorn<br>• Bread Tijger brown wheat<br>• Bread Tijger wholemeal<br>• Bread wholemeal fine<br>• Bread wholemeal average<br>• Bread multigrain wholemeal Becel<br>• Bread VollerKoren<br>• Bread Blue Band Goede Start white bread<br>• Bread white Brinta Vezelwit<br>• Roll brown soft<br>• Roll wholemeal soft<br>• Breakfast cereal Brinta<br>• Crispbread wholemeal<br>• Oatmeal<br>• Wheat germ<br>• Crispbread light<br>• Flour wheat wholemeal<br>• Flour soya full fat<br>• Breakfast cereal All--Bran Plus Kellogg's<br>• Breakfast prod Albona 7--cereals--energy<br>• Breakfast cereal porridge Bambix Zonnig Ontbijt licht volk<br>• Breakfast cereal Weetabix original<br>• Bread rye average<br>• Bread rye dark<br>• Bread rye light<br>• Oat bran raw<br>• Wheat bran<br>• Bread wheatrye wholemeal
Whole-grain products or potatoes	• Potatoes slices/parts frozen unprepared<br>• Potatoes boiled w skin average<br>• Potato waffels/balls frozen unprepared<br>• Potato product natural precooked chilled<br>• Potato product seasoned precooked chilled<br>• Potatoes slices/parts frozen with spices unprepared

- Potato puree powder av
- Potato puree powder wo milkpowder wo fat
- Potato puree powder w milkpowder w fat
- Potatoes wo skins boiled average
- Potato sweet boiled
- Bulgur wheat cooked
- Chips oven frozen prepared
- Barley whole grain raw
- Rice brown boiled
- Pasta wholemeal boiled
- Quinoa cooked
- Rosti prepared wo fat
- Rosti rounds frozen unprepared

Dairy, nuts, fish, legumes, meat and eggs

Fish/ Legumes/meat/eggs

- Anchovy in oil canned
- Anchovy raw
- Anchovy prepared without fat
- Ocean perch prepared in microwave oven
- Kipper smoked
- Beans brown boiled
- Beans brown canned
- Beans kidney red canned
- Beans kidney red boiled
- Beans mung boiled
- Beans black eyed canned
- Beans runner tinned
- Beans runner boiled
- Beans soya boiled
- Beans French tinned
- Beans French boiled
- Beans French frozen boiled
- Beans white canned
- Beans white boiled
- Pea garden super fine tinned
- Peas frozen boiled
- Peas fresh boiled
- Egg whole chicken average boiled
- Egg yolk chicken boiled
- Egg white chicken raw
- Peas chick boiled
- Chickpeas canned
- Splitpeas yellow boiled
- Splitpeas green boiled
- Halibut smoked
- Trout prepared in microwave oven
- Prawns cooked
- Shrimps Dutch peeled boiled

- Mince chicken raw
- Chicken wo skin raw
- Herring salted
- Herring pickled (sweet)sour
- Venison raw
- Squid prepared in microwave oven
- Squid rings battered deep--fried
- Cod boiled
- Whitefish fillet batter deepfried wo fat
- Veal rib steak raw
- Veal stewing steak raw
- Veal frying steak raw
- Turkey fillet raw
- Turkey raw
- Peas marrowfat canned
- Peas marrowfat boiled
- Peas marrowfat young canned
- White fish fillet in batter deep--fried
- Chicken w skin raw
- Chicken drumstick w skin raw
- Chicken fillet raw
- Chicken fillet breaded raw
- Chicken rolled raw
- Rabbit domesticated raw
- Pollock Alaska steamed
- Crab in water tinned
- Surimi
- Lobster boiled
- Spawn/hard roe coloured
- Pheasant whole raw
- Lamb leg raw
- Liver chicken raw
- Liver pork raw
- Lentils brown canned
- Lentils red boiled
- Lentils green and brown boiled
- Mackerel steamed
- Mackerel fillet smoked
- Mackerel prepared in microwave oven
- Mackerel in oil tinned
- Kidney ox raw
- Horse meat raw
- Eel smoked
- Eel prepared in microwave oven
- Pangasius prep in microwave oven
- Mange--tout boiled
- Beef rump steak raw

- Beef <5% fat raw average
- Beef stewing meat raw
- Beef rib raw
- Beef frying steak raw
- Beef streaked/marbled raw
- Beef rib steak raw
- Beef stewing steak raw
- Beef tenderloin steak raw
- Beef ribeye steak raw
- Beef sirloin rolled raw
- Beef roast raw
- Beef shank raw
- Beef for stewing averaged raw
- Beef steak tartare raw
- Sardines/pilchards in oil tinned
- Sardines grilled
- Dab/lemon dab prep in microwave oven
- Pork schnitzel not breaded raw
- Fish fingers unprepared
- Plaice boiled
- Tilapia prepared without fat
- Sole prepared in microwave oven
- Tuna in oil tinned
- Tuna in water tinned
- Tuna raw
- Tuna prepared without fat
- Beans broad tinned
- Beans broad boiled
- Pork fricandeau part of leg raw
- Minced pork raw
- Pork tenderloin raw
- Gammon boiled deboned
- Pork gammon steak raw
- Pork shoulder chop raw
- Pork loin chop raw
- Pork fillet raw
- Pork chop raw
- Pork shoulder raw
- Pork stewing meat raw
- Pork tenderloin medaillon raw
- Pork sparerib raw
- Fish lean 0--5 g fat raw
- Rabbit wild raw
- Meat average raw excl liver
- Beef average raw
- Salmon tinned
- Salmon smoked

	• Salmon farmed raw<br>• Salmon farmed prep in microwave oven<br>• Rainbow trout prepared in microwave oven<br>• Salmon pate/--mousse<br>• Mussels boiled
Unsalted nuts	• Almonds blanched unsalted<br>• Chia seeds dried<br>• Hemp seed<br>• Chestnuts raw<br>• Linseeds<br>• Poppy seeds<br>• Nuts mixed unsalted<br>• Cashew nuts unsalted<br>• Hazelnuts unsalted<br>• Nuts macadamia unsalted<br>• Brazil nuts unsalted<br>• Pecan nuts unroasted unsalted<br>• Walnuts unsalted<br>• Pine nuts<br>• Peanuts unsalted<br>• Peanuts dry roasted<br>• Pumpkin seeds<br>• Sesame seeds<br>• Sunflower seeds
Dairy	• Yoghurt drink w sweetener<br>• Milk chocolate--flavoured w sw Optimel<br>• Yoghurt drink Vifit fruit<br>• Yoghurt drink Yomild drink fruit<br>• Breakfast drink Goede Morgen original<br>• Breakfast drink Goede Morgen fruit<br>• Yoghurt drink w sweeteners Optimel<br>• Yoghurt drink Fristi<br>• Buttermilk curds fresh<br>• Buttermilk<br>• Milk semi--skimmed<br>• Coffee creamer w pufa Becel<br>• Coffee creamer low fat<br>• Coffee creamer Balance<br>• Fromage frais low fat<br>• Yoghurt l fat w fruit/van w sw Optimel<br>• Fromage frais low fat w fruit w sw<br>• Milk chocolate--flavoured w sweetener w inulin<br>• Hot chocolate from vending machine<br>• Milk skimmed<br>• Milk semi--skimmed lactose free<br>• Buttermilk w fruit<br>• Yoghurt drink Topvit w sweetner

	• Milkdrink skimmed milk Becel pro--activ<br>• Milk skimmed dried<br>• Milk based drink Yakult light<br>• Yoghurt drink Becel pro--activ<br>• Yoghurt drink Actimel 0% fat natural<br>• Milkbased drink Yakult plus<br>• Drink soya Original Alpro<br>• Drink soya Light Alpro<br>• Drink soya unsweetened Alpro<br>• Drink soya sweetened Milbona nature<br>• Drink soya unsweetened Milbona/Albert Heijn<br>• Yoghurt low fat<br>• Yoghurt half fat<br>• Yoghurt 0% fat w fruit Activia<br>• Yoghurt Bulgarian low fat<br>• Yoghurt drink Fristi with sweetners<br>• Yoghurt drink w sweeteners enriched w calcium<br>• Yoghurt drink Optimel Puur<br>• Yoghurt low fat with fruit w sweetener<br>• Yogurt low fat Activia
Cheese	• Dairy spread plain/herbs<br>• Cheese 30+ average<br>• Cheese goat fresh<br>• Cheese 30+ age 4--8 weeks<br>• Cheese 20+ Leidse w cumin/Fries clove<br>• Cheese 30+ age 8 wk--4 mths<br>• Cheese 30+ less salt age 4--7 mths<br>• Cheese 20+<br>• Cheese 30+ age 4--7 mths<br>• Cheese 30+ less salt age 8 wk--4 mths<br>• Cheese 30+ less salt age 4--8 weeks<br>• Cheese 30+ age 10--12 mths<br>• Cheese Mozzarella made from cow's milk<br>• Cheeseprod w veg fat Kees extra gerijpt<br>• Cheese Ricotta<br>• Cheese spread Eru Balans<br>• Cheese spread Eru kids<br>• Dairy spread plain/herbs light
**Spreading and cooking fats**	
Spreadable fats and cooking fats	• Margarine liquid 80% fat Vita d'Or Bewust<br>• Low fat margarine prod 35% fat <10 g sat<br>• Low fat margarine prod AH chol verlagend<br>• Low fat margarine prod C1000 Goed begin<br>• Low fat marg prod Becel calorie light<br>• Low fat spread AH omega--3<br>• Low fat margarine product Goed Begin Albert Heijn/Jumbo

- Low fat margarine 40% fat <17g sat w vit E
- Low fat margarine product Vita d'Or Bewust light
- Oil peanut
- Oil Becel
- Oil linseed
- Oil corn
- Oil rice bran
- Oil sesame
- Oil soy
- Oil safflower
- Oil wok
- Oil sunflower seed
- Oil olive
- Frying fat liquid
- Frying fat horeca
- Oil vegetable average
- Low fat margarine product tub Becel Ligh
- Low fat marg 40% fat <17 g sat unsalted
- Oil rapeseed

Drinks

Fluids

- Water average
- Coffee prepared
- Coffee instant powder
- Tea prepared
- Tea herbal instant sw prepared
- Tea herbal instant powder
- Mineral water Bar le Duc
- Mineral water Evian
- Mineral water Vittel

Other products

- Porridge oatmeal
- Porridge rice
- Porrdige buttermilk groats
- Porridge semolina
- Porridge oatmeal w semi--skimmed milk
- Blancmange vanilla
- Crispbakes Dutch farmers cereals&seeds Bolletje
- Bread brown w sunflower seeds
- Bread brown w seeds
- Bread brown w pumpkin seeds
- Bread wholemeal fine w seeds
- Bread corn w sunflower seeds
- Bread corn w seeds
- Bread wholemeal average w pumpkin seeds
- Bread wholemeal average w sunflowerseeds
- Bread wholemeal average w seeds
- Bread wholemeal fine w sunflower seeds

- Bread wholemeal coarse w seeds
- Bread wholemeal coarse w sunflower seeds
- Bread wholemeal coarse w pumpkin seeds
- Mixed nuts and raisins
- Cabbage red glass
- Cabbage red w apple pieces frozen boiled
- Cabbage red w apple pieces glass
- Cabbage red w apple pieces
- Peas and carrots tinned
- Peas and carrots frozen unprepared
- Mange--tout and carrots boiled
- Potato croquettes frozen unprepared
- Strawberries in syrup tinned
- Apricots in syrup tinned
- Advocaat liqueur
- Aioli
- Almond filled pastry
- Drink almond Alpro Original
- Drink almond unsweetened Alpro
- Almonds blanched salted
- Liqueur >25 vol% alcohol
- Pineapple in syrup tinned
- Sugar granulated
- Apple strudel
- Apple turnover
- Apple turnover w puff pastry w butter
- Apple sauce wo sugar w sweetener tinned
- Vegetables mixed pickled Atjar tjampoer
- Gherkins sweet pickled
- Vinegar
- Bacon
- Liqueur with cream 15--25 vol% alcohol
- Cooking fat solid 97% fat >17 g sat
- Cooking fat solid 80% fat >17 g sat
- Margarine 80% fat > 24 g sat unsalted
- Margarine liq 80% fat < 17g sat unsalted
- Cooking fat liq 97%fat <17g sat unsalted
- Cooking fat sol 97% fat>17g sat unsalted
- Margarine product liquid light unsalted
- Margarine product liquid light
- Low fat margarine product Vita d'Or Goed begin
- Margarine liquid 80% fat Becel Olijf
- Cooking fat liq Vita d'Or vlees&jus
- Baklava nut--honey cake
- Scrapple pork
- Chines noodle ball unprepared
- Fritter banana

- Berenburg herb liquor
- Meatball Berenklauw unprepared
- Berliner pastry
- Crispbakes Dutch
- Crispbread sesame
- Crispbakes Dutch wholemeal
- Crispbread Oerknack Bolletje
- Cranberries dries sweetened
- Beer pilsner
- Beer >7 vol% alcohol
- Beer alcohol free <0 1 vol%
- Beer with fruit juice Radler alcohol free
- Beer brown
- Beer bock
- Beer w fruit flavour
- Beer white
- Beer with fruitjuice Radler
- Beetroot pickled glass
- Binding agents averaged
- Biscuit sweet
- Biscuit w dried fruit & yoghurt Yofruit
- Biscuit with chocolate
- Chocolate chip cookie
- Biscuit fortified Bridge milkbiscuit
- Biscuit brown/digestive w chocolate
- Biscuit filled
- Biscuit w chocolate layer Scholiertje
- Biscuit digestive
- Biscuit fortified Provita milkbiscuit
- Biscuit gluten free Glutafin
- Biscuit brown/wholemeal
- Biscuit muesli
- Biscuit fortified Liga Belvita ontbijtbiscuits
- Biscuit average
- Biscuit Cafe noir
- Biscuit fortified LU Time Out
- Jagermeister herb liquor
- Croquette meat Bitterbal prep in oven
- Croquette meat ragout frozen unprep
- Biscuit Dutch Amaretti Bitterkoekjes
- Barley easy cook raw
- Flour rice
- Flour wheat white 75% extraction
- Buckwheat groats
- Herb and vegetable mix prepared
- Mix for rice/Chinese noodles prepared
- Cassava cake Bojo Surinam

- Meringue cake Bokkenpootje
- Beans black canned
- Minced meat w ham and cheese raw
- Peanuts coated
- Fondant cream
- Chocolate eclair
- Herb butter
- Butter product melange salted
- Butter product half fat
- Butter product 25% fat
- Butter salted
- Butter product melange unsalted Campina Botergoud
- Stock portion prepared
- Stock from cube prepared
- Stock powder
- Stock powder low sodium
- Stock cubes
- Brandy
- Roll white soft
- Bread white w sunflower seeds
- Bread white average w seeds
- Bread brioche
- Bread toasted
- Bread multigrain gluten free Rustico
- Bread raisin
- Bread white gluten free Pan Carre Schar
- Bread low in carbohydrates
- Bread currant
- Bread currant w almond paste
- Bread raisin/current average
- Bread current/raisin w almond paste
- Bread corn
- Bread wholemeal w nuts
- Bread brown/wholemeal w muesli
- Bread raisin w almond paste
- Baguette w cheese--onion
- Bread white w sugar Suikerbrood
- Bread wheat malt
- Bread Tijger white
- Bread brown Turkish
- Bread white Turkish
- Bread white average milk/water based
- Bread ciabatta no filling
- Bread white milk based
- Bread Omega--
- Bread white water based

- Roll white hard
- Bread stuffed Bapao meat
- Roll brown hard
- Roll multigrain hard
- Croissant chocolate--
- Snack roll puff pastry with Dutch sausage Frikandel
- Spring roll fried
- Cheese pasty w puff pastry
- Danish pastry
- Bun currant/raisin
- Bread current wholemeal
- Roll multigrain soft
- Bun wholemeal w muesli
- Bread pita white
- Bun with vanilla custard
- Ragout pasty w puff pastry
- Snack sausage roll puff pastry
- Snack sausage roll w bread dough pastry
- Cream slice Dutch Tompouce
- Flan hard shell w custard cream
- Biscuits sugar free
- Brownie without filling
- Crisps maize Bugles
- Chicken nuggets prepared in oven
- Pork schnitzel sate breaded raw
- Cocoa powder
- Cocoa product sweetened Nesquik Hot Choc
- Cocoa powder sweetened Nesquik
- Cake wo butter
- Cake chocolate made w butter
- Gateau w whipped cream
- Cake apple--
- Cup cake iced
- Cake with nuts
- Gateau fatless sponge w marzipan
- Cake made w butter and apple
- Cake chocolate made wo butter
- Cake made w butter
- Cake marble--
- Cake Indonesian
- Campari
- Candybar Mars
- Candybar Snickers
- Candybar Twix
- Pork side cured and smoked
- Salami sausage saveloy
- Wine white dry

- Cocktail snacks Nibb--it
- Crisps based on potato flour
- Cocktail snacks based on corn or wheat
- Crisps potato unflavoured
- Crisps potato flavoured
- Cocktail snacks Wokkels
- Crisps potato light unflavoured
- Potato crisps oven baked
- Crisps potato light flavoured
- Crisps potato straws flavoured
- Crisps potato Lays Sensations flavoured
- Crisps tortilla several flavours
- Crisps potato average
- Chocolate dark
- Chocolate milk
- Chocolate bar milk w nuts
- Chocolate white
- Chocolate filled with caramel Rolo
- Chocolate extra dark
- Maltesers
- M&M's chocolate
- Candybar Bounty
- Chocolate liqueurs
- Chocolates filled/Belgium chocolate
- After eight chocolate mints
- Chocolate milk w raisins
- Chocolate dark w nuts
- Chocolate milk w puffed rice
- Candybar KitKat
- Chocolate bar filled Kinder
- Chocolate bar milk without sugar
- Chocolate butter
- Spread chocolate hazelnut
- Spread chocolate dark
- Spread chocolate milk
- Spread duo w chocolate
- Chocolate spread white
- Biscuit chocolate coated
- Mango chutney
- Baguette brown
- Cider
- Cognac
- Fruit in syrup tinned
- Cranberry compote sweetened
- Biscuit w nuts and chocolate
- Biscuit oatmeal
- Biscuit fruit

- Pork filled with ham and cheese raw
- Chicken cordon bleu raw
- Corned beef
- Breakfast cereal Cornflakes Kellogg's
- Breakfast cereal All--Bran Fruit n Fibre
- Breakfast cereal Special K Original
- Breakfast cereal Honey pops Loops
- Breakfast cereal Cornflakes
- Muesli crunchy plain/w fruit
- Corn flakes Golden Bridge
- Breakfast cereal Spec K choc Kellogg's
- Breakfast cereal Tresor Kellogg's
- Breakfast cereal cornflakes Plus/1 de Beste
- Breakfast cereal Chocoschelpjes Perfekt/Markant
- Couscous boiled
- Crackers cream
- Crispbread averaged
- Crispbread gold--brown
- Crispbread high fibre
- Cracker mini unflavoured
- Crispbread Cracottes
- Cracker mini flavoured
- Crisp bread gluten free Fette Croccanti
- Crackers VitaLU
- Toast Melba other varieties
- Crispbread wholemeal Cracottes
- Crispbread Cracottes Vital
- Cracker VitaLU w added calcium
- Biscuits & snacks cheesy
- Pudding airy
- Creme fraiche
- Creme fraiche half fat
- Butter cream
- Croissants
- Croissant prepared w butter
- Croissant prepared wo butter
- Croissant average
- Croissant ham and cheese
- Croissant cheese
- Croutons
- Sweets boiled
- Puff pastry baked
- Puff pastry w butter baked
- Filopastry unprepared
- Sauce chocolate for pudding
- Sauce fruit for pudding
- Saus caramel for pudding

- Dfr Modifast Intensive Milkshake
- Pd Nutridrink Compact p 100 ml Nutricia
- Pd Nutridrink Compact Protein p 100 ml
- Pd Fresubin energy drink p 100 ml
- Pd Fresubin jucy drink
- Pd Fresubin protein energy drink p 100ml
- Modular protein powder Protifar Nutricia
- Modular preparation Fantomalt
- Meal replacer milkshake powder Herbalife
- Fruit juice concentrated
- Fruit juice concentrated prepared
- Doughnut iced
- Doughnut plain
- Fruit juice drink ACE average
- Fruit juice drink ACE Aldi/Kruidvat
- Fruit juice drink diary Wicky fruitzacht
- Fruit juice dk ACE Fruxano/Super/Tasting
- Breezer
- Gin young Dutch
- Soft drink cola w caffeine
- Sports drink AA High Energy
- Sports drink Extran Energy
- Energy drink Golden Power/Bullit/Freeway
- Sports drink Aquarius
- Energy drink Red Bull
- Energy drink Red Bull sugarfree
- Energy drink Mixxed up
- Soft drink light wo caffeine
- Sports drink AA Isotone
- Sports drink Extran Hydro
- Sport drink River Powerdrink/Freeway
- Whey drink Taksi w sugar
- Whey drink Taksi w sweetener
- Whey drink
- Juice orange pasteurized
- Juice drink
- Fruit juice dk minimal 2 fruits
- Juice drink light
- Fruit juice drink Roosvicee Multivit
- Juice drink Dubbelfrisss
- Fruit juice dk Coolbest skinny orange
- Juice drink Wicky
- Fruit juice dk Roosvicee Original/frdr
- Juice multifruit
- Fruit juice dk minimal 2 fruits w vit C
- Juice Drink Ocean Spray
- Juice drink w sugar & sw Wicky

- Fruit juice drink apple nectar
- Juice multifruit-- with vitamin E Healthy People
- Fruit juice drink juice and water
- Breakfast drink HeroFruitontbijt p 100ml
- Juice drink Tintelfruit w vit C
- Juice drink Dubbelfrisss light
- Juice drink Spa&Fruit still
- Juice drink Spa&Fruit light still
- Juice fruit Multi Vitamientje oranje vr
- Fruit drink Multi Vitamientje bosvr
- Juice fruit Coolbest Vitaday original
- Juice drink w sugar & sw
- Juice drink multivitamin Capri--Sun
- Fruit juice drink 12 fruits nectar light
- Lemonade fruit Wicky zero
- Fruit juice drink Fruity King tropical
- Fruit juice dk Surango multivit light
- Fruit juice dk Solevita multivit 12 fru
- Fruit juice dk Roosvicee Multivit light
- Fruit drink Roosvicee 50/50
- Fruit drink Linessa Vital&Active light
- Juice drink Spa&Fruit sparkling
- Vitaminwater
- Salad cream 25% oil
- Salad dressing naturel wo oil
- Salad dressing Yofresh
- Salad dressing vinaigrette
- Salad dressing 20% oil w yoghurt
- Mayonnaise
- Sauce for chips approx 13% oil
- Salad dressing honey/mustard
- Sauce for chips 5% oil
- Sauce Joppie
- Salad dressing Becel Light
- Sandwich spread original
- Mayonnaise yoghurt based 25% oil
- Salad dressing olive oil--vinegar
- Breakfast drink Ontbijt&Fit
- Yoghurt drink
- Dairy drink Milk&Fruit original
- Liquorice Dutch type sweet
- Liquorice Dutch type average
- Liquorice sugarfree
- Liquorice Dutch type double salted
- Liquorice allsorts
- Liquorice w peppermint
- Liquorice Dutch type sweet with foam

- Liquorice Dutch type salted
- Mentos cheewy dragee
- Duck whole raw
- Biscuit fortifd w currants LigaEvergreen
- Biscuit fortified Bridge fruit and grain
- Biscuit fortified Evergreen crunchy
- Falafel unprepared
- Beef steak tartare spiced filet americ
- Liqueur <15 vol% alcohol
- Focaccia
- Pork fricandeau fried
- Sausage Frikandel deep--fried liquid fat
- Sausage Dutch Frikandel frozen unprep
- Soft drink wo caffeine
- Ice tea with sugar and sweetener
- Sport drink Freeway Sportivo
- Soft drink w sugar & sweetener 2--<5 g KH
- Cola light soft drink w caffeine
- Soft drink w sugar sw&caffeine 2--<5 carb
- Softdrink w sugar sw&caffeine 5--<8g carb
- Ice tea
- Juice drink Tintelfruit light
- Ice tea light
- Ice tea with less sugar
- Lemonade fruit Sisi no bubbles Action
- Whey drink Rivella wo sugar
- Chips pre--fried frozen unprepared
- Chips prepared average
- Sauce for chips 25% oil
- Mayonnaise product approx 35% oil
- Sauce garlic 20--<30% oil
- Crisps potato straws natural
- Fruit cocktail in syrup tinned
- Biscuit fortified Liga Evergr assortment
- Sweets jelly/gums/foam
- Sweet fruity chew
- Forcemeat balls for soup canned
- Minced beef raw
- Minced beef/pork raw
- Minced meat raw average
- Minced meat loaf fried
- Minced beef/pork ball prep w egg/crumbs
- Minced beef ball prepared without egg
- Minced meat beef/pork raw w egg/brcrumbs
- Minced meat (50:50 pork beef) w veg raw
- Minced meat pork raw w egg and breadcrumbs
- Jam

- Ginger stem in syrup tinned
- Millet boiled
- Yeast extract Marmite
- Icing (sugar)
- Guacamole
- Coloured confetti fruit--flavoured
- Chocolate confetti milk
- Chocolate confetti averaged
- Chocolate confetti dark
- Chocolate confetti white
- Chocolate confetti mix white and dark
- Chocolate confetti extra dark
- Margarine product tub Becel Dieet
- Low fat spread Becel pro--activ
- Low fat marg prod Blue Band Goede Start
- Low fat marg prod 20--25% fat <10 g sat
- Margarine product 70% fat <17 g sat
- Margarine product 70% fat >17 g sat
- Margarine product 60% fat <17 g sat
- Low fat margarine Gouda's Glorie Lichtste Pondje
- Low fat margarine prod Becel Omega3 Plus
- Low fat margarine prod Blue Band Idee
- Low fat margarine prod Bewust light
- Margarineprod AlbertHeijn Bewust Balans
- Low fat marg prod Dieetella Double Activ
- Low fat margarine product Gezonde Start
- Low fat spead Becel pro.activ light
- Margarine product tub Jumbo diet
- Margarine product 60% fat >17 g sat
- Margarine product Vita d'Or Bewust
- Margarine product Jumbo bewust
- Ham lean boiled
- Ham smoked raw
- Ham lean grilled
- Ham shoulder medium fat boiled
- Hamburger raw
- Fromage frais full fat w fruit
- Rice drink
- Liquorice Stophoest
- Boiled sweets sugar free
- Honey
- Sandwich spread other flavours
- Hummus natural
- Cheese cottage
- Ice cream dairy cream based
- Ice cream stracciatella--
- Ice cream dairy w chocolate coating

- Ice cream dairy w fruitcoating
- Icecream based on coconutmilk
- Ice lolly
- Ice lolly Festini
- Ice cream dairy cornet
- Icecream with caramel and nuts
- Sorbet
- Fruit drink conc diluted average
- Jam rose hip
- Jam reduced sugar
- Marmalade
- Biscuits w nuts
- Japanese rice cracker mix w peanuts
- Japanese rice cracker mix wo peanuts
- Gin Dutch red currant flavoured
- Gin Dutch lemon flavoured
- Gin old Dutch
- Biscuit children's average
- Biscuit Dutch Jodekoek
- Gravy 25% fat thickend prep w gravypowde
- Gravy 25% fat thick prep wo gravy powder
- Gravy 25% fat thickend w gravypowder
- Gravy 25% fat w margarine liquid 2558
- Gravy 25% fat w cooking fat liquid 2562
- Gravy 25% fat w margarine 2063
- Gravy 25% fat clear prep wo gravy powder
- Gravy 25% fat w butter
- Gravy 25% fat thickend w marg 2063
- Gravy 25% fat thickend w cook fat 2067
- Gravy 25% fat thickend w olive oil
- Gravy 25% fat w marg 2063 + gravy powder
- Gravy 25% fat w marg 2077 w gravypowder
- Gravy 25% fat w marg 2558 + gravy powder
- Gravy 25% fat clear prep w gravy powder
- Gravy 25% fat w cooking fat 2067
- Gravy 25% fat w margarine liquid 2077
- Gravy 25% fat w olive oil
- Gravy 50% fat thick prep wo gravypowder
- Gravy 50%fat thickend prep w gravypowder
- Gravy 50% fat clear prep wo gravypowder
- Gravy 50% fat w cooking fat 2067
- Gravy 50% fat w margarine 2063
- Gravy 50% fat w margarine liq 2077
- Gravy 50% fat w margarine liquid 2558
- Gravy 50% fat w cooking fat liquid 2562
- Gravy 50% fat w cooking fat 2563
- Gravy 50% fat w olive oil

- Gravy 50% fat(butter) prep wo gravypowd
- Gravy 75% fat thick prep wo gravypowder
- Gravy 75%fat thick prep w gravy powder
- Gravy 75% fat clear prep wo gravypowder
- Gravy 75% fat w margarine 2063
- Gravy 75% fat w cooking fat 2067
- Gravy 75% fat w margarine liq 2077
- Gravy 75% fat w olive oil
- Gravy 75% fat w cooking fat 2563
- Gravy 5% fat thick prep wo gravypowder
- Gravy 5% fat thickend w gravy powder
- Gravy 5% fat clear prep wo gravypowder
- Gravy no fat prep w gravy powder
- Cheese Gouda 48+ average
- Cheese Saint Paulin/Port Salut
- Cheese Gruyere
- Cheese Emmenthaler
- Cheese 48+ less salt average
- Cheese Rambol
- Cheese raw milk 48+
- Cheese Gouda 48+ age 4--7 mths
- Cheese sheep/goat Turkish 50+ canned
- Cheese Roquefort
- Cheese Bluefort
- Cheese Gorgonzola
- Cheese 45+
- Cheese spread 48+
- Cheese Brie 50+
- Cheese Camembert 30+
- Cheese Brie 60+
- Cheese Camembert 45+
- Cheese Cheddar
- Cheese Edam 40+
- Cheese sheep fresh
- Cheese white 45+ feta--like from cow's milk
- Cheese Dutch in Swiss--style 45+
- Cheese goat hard
- Cheese 50+
- Cheese 40+ Leiden w cumin/Fries clove
- Cheese 10+
- Cheese Gouda 48+ age 8 wk--4 mths
- Cheese 48+ less salt age 4--7 mths
- Cheese Parmesan
- Cheese 48+ less salt age 8 wk--4 mths
- Cheese Gouda 48+ age 4--8 weeks
- Cheese 48+ less salt age 4--8 weeks
- Cheese Old Amsterdam 48+

- Cheese Gouda 48+ age 10--12 mths
- Cheesespread Heks'n kaas
- Mascarpone cheese
- Cheese cream soft Boursin
- Cheese smoked
- Cheese cream soft Mon Chou
- Cheese cream 60+
- Cheese cream soft Paturain
- Cheese cream with herbs 25--30 g fat
- Cheese spread 40+
- Cheese spread 20+
- Cheese spread 30+
- Cheese spread 60+ Kiri
- Cheese spread 45+
- Cheese Stilton
- Cheese Swiss dried
- Veal olive raw
- Minced veal raw
- Biscuit sponge fingers
- Fudge/caramel soft
- Toffees
- Chewing gum
- Chewing gum wo sugar
- Yoghurt vanilla half fat
- Cherries in syrup tinned
- Biscuits assorted w butter
- Stollen w almond/imitat paste average
- Stollen w almond/imitat paste w nuts
- Stollen w almond/imitat paste wo nuts
- Ketchup hot
- Ketchup curry
- Salsa tomato dip
- Ketchup tomato
- Soya sauce salt
- Frogs/mice fondant with chocolate
- Chicken fillet in batter raw
- Sandwich meat chicken
- Chicken for soup w skin raw
- Chicken sticks breaded
- Chicken schnitzel raw
- Biscuit Dutch Kletskop
- Crispbread Sandwich Wasa
- Fruit snack Knijpfruit/Slurpfruit
- Biscuit shortbread Bastogne
- Cake butter Dutch Boterkoek
- Nuts cake
- Cake sponge Dutch Eierkoek

- Sponge cake wholemeal
- Almond paste filled tarts w butter
- Almond paste filled tarts wo butter
- Biscuit Jaffa cakes/Cake PiM's
- Tarts filled w jam
- Coconut flavoured cookies
- Cake Dutch spiced Ontbijtkoek w raisin
- Cake Dutch spiced Ontbijtkoek w nuts
- Cake Dutchspiced Ontbijtkoek w rockcandy
- Cake Dutch Ontbijtkoek w nuts&fruit
- Cake Dutch spiced Ontbijtkoek
- Cake Dutch spiced Ontbijtkoek w ginger
- Cake Dutch spiced Ontbijtkoek less sugar
- Cake Dutch spiced Lekkere Start
- Cake Dutch spiced ontbijtkoek w chocolate
- Cake Dutch spiced Ontbijtkoek wholemeal
- Cake Dutch w icing & cream 'Oranjekoek'
- Biscuit peanut
- Shortbread
- Cereal bar w milk Kellogg's
- Biscuit Dutch shortbread sprits
- Biscuit Dutch Frou frou
- Biscuit salted average
- Coffee w milk vending machine
- Coffee from vending machine with sugar
- Coffee w sugar and milk vending machine
- Coffee Cappuccino freshly made
- Coffee cappuccino instant prepared
- Coffee iced
- Coffee wiener melange instant prepared
- Coffee cappuccino instant powder
- Coffee wiener melange instant powder
- Coffee latte macchiato freshly made
- Coffee creamer full fat
- Coffee creamer half fat
- Coffee creamer 20% fat
- Coffee creamer powder
- Coffee creamer low fat powder
- Coconut bread sweetened sliced
- Coconut macaroons
- Cucumber sliced pickled
- Cabbage sauerkraut cooked
- Biscuit Dutch Krakeling
- Prawn crackers natural
- Cassave crackers
- Croquette meat ragout prepared in oven
- Biscuit spiced small

- Biscuit spiced small Kruidnoten w dark choc
- Biscuit spiced small Kruidnoten w milk choc
- Biscuit spiced small Kruidnoten w white choc
- Fromage frais full fat
- Fromage frais half fat w fruit
- Fromage frais low fat with fruit
- Fromage frais half fat
- Fromage frais w fruit Danoontje
- Cake raisins--
- Fromage frais yoghurt w fruit
- Lamb chop raw
- Lamb shoulder raw
- Liver ox boiled
- Liver pork boiled
- Liver pate/Berliner liver sausage
- Liver pate
- Pate spreadable lean
- Biscuit fort Liga 12--36 mnth
- Biscuit fortified Liga Continue vit/min
- Biscuit fortified Liga Haverkick
- Biscuit fortified Liga Milkbreak milkbiscuit
- Biscuit fortified AH/Jumbo/Aldi
- Biscuit fortified Liga Baby 6--12 mnth
- Biscuit fortified Liga Baby 4--6 mnth
- Candybar Lion
- Springroll frozen unprepared
- Liquorice w sal ammoniac powder
- M&M's chocolate w peanuts
- Meal replacer mlkshk prep s--sk milk
- Meal replacer Cambridge shake/soup prep
- Pd Nutridrink Multi Fibre per 100 ml
- Cereal bar Hero B'tween free
- Meal replacer bar Weightcare
- Cornflour
- Tangerines in syrup tinned
- Marzipan
- Marsh mallows
- Crackers matzes
- Sauce for chips 35% oil
- Mayonnaise product w olive oil
- Starch potato
- Flour wheat self--raising
- Flour buckwheat
- Cornmeal
- Bread crumbs
- Milk chocolate--flavoured full fat
- Milk chocolate--flavoured low fat

- Milk chocolate--flavoured semi--skimmed
- Drinking chocolate w s--sk milk
- Drinking chocolate w s--sk milk and Nesquik
- Milk goats-- full fat
- Milk raw
- Milk whole
- Milkshake
- Dairy drink Campina fruitmilk
- Milk whole dried
- Cake wrapped in marzipan and chocolate
- Pasta white average boiled
- Noodles boiled
- Milkshake McDonald's
- Candybar Milky Way
- Milk based drink Yakult original
- Yoghurt drink Actimel natural
- Yoghurt drink Actimel fruit
- Miso soya paste
- Flour for pancakes
- Flour for pancakes multigrain
- Curry powder djawa
- Herb and dried vegetable mix
- Mixed spices Wereldgerechten unprep
- Mix rice/Chines noodles unprepared
- Mix Maggi oven/dagschotel unprepared
- Mix seasoning Mexican unprepared
- Sauce powder approx 30% fat
- Mix for marinade powder unprepared
- Mix Hollands Pannetje Maggi unprepared
- Sauce powder approx 10% fat
- Mustard
- Mousse chocolate
- Custard w whipped cream
- Coconut milk
- Muesli crunchy w nuts
- Muesli w fruit
- Muesli Country Store Kellogg's
- Muesli crunchy w chocolate
- Muesli crunchy w nuts and chocolate
- Muesli crunchy Cruesli Balans
- Breakfast cereal Choco moons Crownfield
- Sports food energy bar Isostar
- Muesli bar w chocolate
- Cereal bar Hero B'tween
- Muesli bar
- Tart w bread base w veg/egg/cheese
- Aniseed comfits crushed/pink&white/blue&white

- Dough for pizza and savoury pie
- Wrap/Tortilla
- Noodles instant prepared
- Nuts mixed salted
- Cashew nuts salted
- Nuts macadamia salted
- Pecannuts oil roasted salted
- Pistachio nuts salted
- Walnuts salted
- Nougat
- Nougat w chocolate
- Oil coconut
- Doughnut Dutch style w currants raisins
- Doughnut Dutch style plain
- Olives average in brine tinned/glass
- Olives green in brine tinned/glass
- Olives ripe in brine tinned/glass
- Breakfast cereal All--Bran flakes
- Breakfast cereal Choco chocos Plus
- Breakfast cereal Coco pops Kellogg's
- Breakfast cereal Honey pops Kellogg's
- Breakfast cereal Honey hoops Crownfield
- Breakfast cereal honey loops 1 de Beste
- Drink breakfast Brinta Wake Up
- Breakfast cereal porridge 8 cereals with honey Bonbebe
- Cocoa product powder Ovomaltine
- Spread duo wo chocolate
- Tortellini boiled
- Pasta gluten free cooked Schar
- Pate
- Sausage luncheon meat
- Pears in syrup tinned
- Beef salted cooked
- Wafer w milk & hazelnts Knoppers
- Waffle penny--
- Peppermint
- Peaches in syrup tinned
- Pesto green
- Pesto red
- Peanut butter
- Peanut butter light
- Peanut butter w nut pieces
- Peanuts salted
- Peanuts milkchocolate coated
- Peanuts sugar coated
- Popcorn popped wo oil natural

- Popcorn popped wo oil sweet
- Popcorn popped wo oil w salt
- Port wine
- Pudding chipolata
- Chocolate blancmange w sauce
- Cream whipped w added sugar
- Dairy dessert with cream averaged
- Blancmange raspberry w red currant sauce
- Blancmange semolina w red currant sauce
- Blancmange home--made w semi--sk milk
- Blancmange chocolate
- Blancmange vanilla w strawberry sauce
- Rhubarb puree w sugar
- Lard
- Rice white boiled
- Rice multi--grain boiled
- Rice white boiled with candied fruit nuts and seeds
- Rice drink w calcium and vitamines
- Rice cakes puffed with salt
- Rice cakes with spices
- Rice cakes puffed with chocolate
- Rice cakes puffed with caramel
- Beef rolled prepared
- Chicken rolled prepared
- Pork rolled (processed meat)
- Beef smoke--dried
- Beef smoke--dried lightly salted
- Horse meat smoked
- Cream whipping
- Cream based on veg oil Alpro Cuisine
- Cream cooking
- Cream cooking light
- Cream type prod Blue Band Finesse
- Cream based on vegoil Alpro CuisineLight
- Cream whipped w sugar canned
- Cream whipped low fat w sweetener canned
- Cream whipped low fat w sugar canned
- Cream sour
- Beef roast prepared
- Roti Surinam pancake
- Raisins coated with milkchocolate
- Rum
- Beef fat
- Beef olives raw
- Salad fish
- Salad ham and leek
- Salad chicken curry

- Salad egg
- Salad cucumber
- Salad meat
- Salad crab
- Salad cheese
- Salad coleslaw
- Salad shrimp
- Salad chicken sate
- Salad celeriac
- Salad tuna
- Salad Russian
- Salade salmon
- Sausage dry salam Turkish
- Salami
- Salami sausage saveloy lean
- Pepper red hot paste
- Pepper red hot fried paste
- Coconut creamed block Santen
- Juice pineapple
- Juice apple
- Juice apple w vitamin C
- Juice redcurrant
- Juice lemon
- Juice grape
- Juice grapefruit
- Juice pear
- Juice orange w pulp
- Juice orange with calcium Appelsientje
- Juice orange Appelsientje Groeifruit
- Juice orange freshly squeezed
- Tomato juice
- Juice tomato Appelsientje Zontomaat
- Juice tomato/vegetable
- Juice tomato/vegetable Appelsientje
- Juice multifruit mild w vit C
- Fruit drink 'portiefruit'
- Juice fruit Coolbest Vitaday tropical
- Juice fruit Solevita multivit 12--fruits
- Juice fruit Hero Fruit&Co
- Juice fruit Vruchtoase multivit
- Sauce tomato based shashlik
- Sauce mix packet <3% fat prepared
- Sauce chilli
- Sauce mix packet >3% fat prepared
- Sauce oriental ready--made in jar/bag
- Sauce hot liquid ready made <12% fat
- Sauce hot liquid ready made >12% fat

- Sauce Aardappel Anders average
- Sauce barbecue
- Sauce mayonnaise based average
- Sauce based on roux prepared
- Sauce butter--
- Seasoning flavoured liquid
- Sauce Chicken Tonight high fat varieties
- Sauce cocktail/party/table 25% oil
- Sauce cocktail/party/table >25% oil
- Sauce garlic 30--<40% oil
- Soya sauce sweet
- Herb paste boemboe
- Sauce tomato ready made jar
- Sauce bolognese ready made jar
- Sauce cheese--
- Sauce soy
- Sauce Chicken Tonight low fat varieties
- Mayonnaise low fat 40% oil
- Sauce prep w s--sk milk en marg 2063/2062
- Sauce oyster
- Piccalilly
- Peanut sauce ready to eat
- Peanut sauce homemade w water w fat
- Peanut sauce packet prepared
- Peanutsauce homemade w s--sk milk wo fat
- Peanutsauce homemade w water milk wo fat
- Sauce chasseur
- Mix Maggi oven--/dagschotel prepared
- Sauce worcester--
- Chicken schnitzel satey breaded raw
- Pork schnitzel breaded raw
- Spice biscuit sprinkles Bolletje
- Wine gum/ fruit gum
- Wine gums with foam layer
- Meringue
- Solid foamed candy 'schuimpje'
- Teacakes chocolate coated marshmellow
- Seitan
- Sherry
- Wrap shoarma roll prep wo fat
- Syrup maple
- Syrup ginger
- Fruit drink concentrate undiluted
- Fruit drink conc w vit
- Fruit drink conc light diluted average
- Fruitdrink conc KarvanCevitam diluted av
- Fruit drink conc w sugar & sw diluted

- Fruit drink conc w vit diluted
- Fruit drink concentrate Roosvicee Origin
- Fruit drink conc Roosvicee Low Cal
- Fruit drink concentrate Karvan Cevitam
- Fruit drink conc Roosvicee Multivit
- Fruit drink conc w sugar and sweetners 40--45g CHO
- Fruit drink concentrate light
- Fruit drink conc can Albert Heijn
- Fruitdrink conc w sugar & sw 10--15 g CHO
- Fruit drink conc w sugars and sweeteners
- Fruit drink conc w 45--50 mg vit C
- Fruit drink concentrate with sweeteners Karvan Cevitam
- Fruit drink conc w sugar diluted AH
- Fruit drink conc diluted 1 to 4
- Fruit drink conc diluted 1 to 7
- Fruitdrink conc w sug&sw diluted Tasting
- Fruit drink concentrated Vruchtenmix diluted
- Fruit drink conc w sugar and sweeteners diluted
- Fruit drink conc with sweetener Karvan Cevitam diluted
- Fruit drink concentrated Vruchtenmix
- Fruit drink conc Roosvicee diluted av
- Syrup from tinned fruit
- Luncheon meat tinned
- Sausage luncheon meat lean
- Sweets cream hard
- Soup instant powder
- Breadsticks
- Eclair filled w banana and whipped cream
- Eclair without filling
- Eclair w whipped cream filling
- Dessert soya Alpro
- Soya based yoghurt w fruit/vanilla Alpro
- Soya based yoghurt natural Alpro
- Drink soya natural
- Drink soya several flavours Alpro
- Biscuit spiced Speculaas w butter
- Biscuit spiced Speculaas wo butter
- Biscuit spiced Speculaas w almond paste
- Spread speculaas flavoured
- Bacon rasher streaky
- Bacon lean smoked raw
- Bacon smoked Katenspek
- Bacon lean smoked prepared
- Bacon fat raw

- Bacon rashers streaky grilled
- Sweets marshmellow type Spekkie
- Spiced cake Indonesian-- Spekkoek
- Almond/imitation paste average
- Spinach creamed frozen boiled
- Biscuit Dutch shortbread sprits w choc
- Baguette white
- Baguette white w herb butter retail
- Syrup apple
- Mineral water w sweetener
- Golden syrup pourable
- Syrup apple--pear
- Syrup apple enriched w iron
- Syrup Keukenstroop
- Syrup sugar
- Waffle syrup average
- Waffle syrup w butter
- Waffle syrup w/o butter
- Biscuit syrup w chocolate
- Cocoa powder sweetened
- Sugar castor brown
- Sugar castor white
- Preparation glucose powder Dextro--M
- Sugar powdered
- Sugar demerara
- Sugar light
- Biscuit savoury Sultana Crunchers
- Biscuit Dutch Taai--taai
- Apple pie Dutch w shortbread w marg
- Appel pie Dutch w shortbread w butter
- Pie apple--nuts
- Chocolate pastry w whipped cream
- Cheesecake made w fromage frais
- Cheesecake made w cream cheese
- Gateau with butter--cream filling
- Meringue w butter--cream
- Gateau fatless sponge w fruit & cream
- Tart fruit w shortbread base
- Crisps tortilla unflavoured
- Sesame paste tahin
- Tahoe soya curd
- Quorn pieces unprepared
- Tapenade olive
- Tapenade tomato
- Tempeh fermented soya beans
- Tea vending machine with sugar
- Tiramisu

- Toast Melba natural
- Toffee w chocolate
- Tomatoes dried in oil tin/glass
- Tomato puree concentrated tinned
- Topping dessert w sugar whipped KlopKlop
- Turkish Delight
- Tzatziki
- Silver--skin onion sweet pickled glass
- Onions deep--fried sachet
- Pork rib chop raw
- Pork spare rib raw
- Bacon rasher raw
- Kromesky meat filled raw
- Sausage luncheon meat vegetarian
- Ham vegetarian
- Vegetable paste Tartex
- Pate vegetarian
- Mincemeat vegetarian unprepared
- Hamburger vegetarian unprep
- Burger vegetarian cheese unprepared
- Beans baked in tomato sauce canned
- Quorn southern style burger unprepared
- Vegetarian schnitzel Valess unprepared
- Vegetable burger vegetarian unpr
- Nuggets vegetarian
- Vegetarian mincemeat balls unprep
- Vegetarian minced meat ball unprepared De Vegetarische Slager
- Pastry puff cheese filled unprepared
- Schnitzel vegetarian unprep
- Vegetarian sausages unprepared
- Vermouth
- Margarine 80% fat < 24 g saturates
- Cooking fat liquid 97% fat <17 g sat
- Frying oil
- Margarine liq 80% fat <17 g saturates
- Low fat margarine 40% fat <17 g sat
- Margarine 80% fat > 24 g saturates
- Frying fat solid
- Butter unsalted
- Margarine liquid 80% fat Blue Band
- Viandel unprepared Mora
- Brandy Dutch vieux
- Custard chocolate full fat
- Custard several flavours full fat
- Custard vanilla full fat
- Custard vanilla low fat

- Custard no fat w sweetener Optimel
- Custard half fat all flavours
- Custard vanilla full fat w choc balls
- Custard soft & airy Campina
- Flan apple and crumble topping
- Flan w custard and crumble topping
- Flan fruit and crumble topping
- Flan filled w rice pudding
- Flan sponge w custard cream
- Flan w fruit filling
- Flan w custard and fruit
- Flan filling tinned
- Custard 2 flavours w syrup vlaflip
- Dessert made of custard yoghurt & syrup
- Pork 5--14% fat raw
- Veal <5% fat raw average
- Lamb >10 g fat raw average
- Ham beef pastirma Turkish
- Minced beef raw w egg and breadcrumbs
- Minced lamb raw
- Veal stewing meat raw
- Pork shoarma seasoning raw
- Veal fricandeau prepared
- Processed meat prod excl liver average
- Sausage excl liver average
- Chocolate flakes milk
- Chocolate flakes dark
- Chocolate flakes mix dark and white
- Chocolate flakes white
- Sweets fruit pectinbased
- Waffle Luikse
- Waffle soft--/sugar--/flash--
- Waffle Luikse w chocolate
- Wafer galette
- Mineral water Spa
- Mineral water average
- Mineral water Chaudfontaine
- Mineral water Sourcy
- Porridge barley w raisins Bessola
- Whisky
- Wine red
- Muscatel
- Wine white sweet
- Wine rose
- Wine gum w liquorice
- Vodka
- Liqueur 15--25 vol% alcohol

- Sausage dried Metworst
- Sausage cooked
- Sandwich meat turkey
- Sausage beef Braadworst raw
- Sausage pork Braadworst raw
- Sausage Chorizo
- Sausage grill
- Sausage grill chicken
- Sausage grill w cheese
- Liver sausage coarse hausmacher
- Sausage frankfurter tinned
- Sausage beef frankfurter type tinned
- Liver sausage
- Sausage raw beef
- Sausage w smoked bacon--bits
- Sausage smoked cooked average
- Sausage smoked lean cooked
- Sausage smoked beef cooked
- Sausage smoked pork cooked
- Liver pate sausage
- Sausage spiced and smoked
- Sausage tongue
- Yoghurt full fat
- Yoghurt low fat w fruit
- Yoghurt full fat w fruit
- Yoghurt snack Breaker
- Yoghurt half fat w fruit
- Yoghurt full fat w fruit/vanilla Activia
- Yoghurt Bulgarian whole milk
- Yoghurt Greek full fat
- Yoghurt drink mini probiotic Optifit
- Yoghurt full fat natural Activia
- Yoghurt drink enriched w calcium
- Yoghurt cream-- w fruit
- Yoghurt full fat w cereal/muesli Activia
- Yoghurt full fat stracciatella
- Yoghurt w fruit Mona Boordevol
- Yoghurt Turkish 4% fat
- Yoghurt Turkish 10% fat
- Seaweed kelp raw
- Seaweed nori dried
- Salty puff pastry cocktail snacks
- Pretzel sticks
- Brawn pork pickled in vinegar
- Bread sourdough wholemeal
- Yoghurt & custard Campina
